# Frequency-specific activation of the peripheral auditory system using optoacoustic laser stimulation

**DOI:** 10.1038/s41598-019-40860-8

**Published:** 2019-03-12

**Authors:** Patricia Stahn, Hubert H. Lim, Marius P. Hinsberger, Katharina Sorg, Lukas Pillong, Marc Kannengießer, Cathleen Schreiter, Hans-Jochen Foth, Achim Langenbucher, Bernhard Schick, Gentiana I. Wenzel

**Affiliations:** 1 Saarland University, Faculty of Medicine, Department of Otolaryngology, Kirrbergerstr. 100, 66421 Homburg, Germany; 2 University of Minnesota, Department of Biomedical Engineering, Department of Otolaryngology, Minnesota, USA; 3 Technische Universität Kaiserslautern, Department of Physics, Kaiserslautern, Germany; 4 Saarland University, Experimental Ophthalmology, Homburg, Germany

## Abstract

Hearing impairment is one of the most common sensory deficits in humans. Hearing aids are helpful to patients but can have poor sound quality or transmission due to insufficient output or acoustic feedback, such as for high frequencies. Implantable devices partially overcome these issues but require surgery with limited locations for device attachment. Here, we investigate a new optoacoustic approach to vibrate the hearing organ with laser stimulation to improve frequency bandwidth, not requiring attachment to specific vibratory structures, and potentially reduce acoustic feedback. We developed a laser pulse modulation strategy and simulated its response at the umbo (1–10 kHz) based on a convolution-based model. We achieved frequency-specific activation in which non-contact laser stimulation of the umbo, as well as within the middle ear at the round window and otic capsule, induced precise shifts in the maximal vibratory response of the umbo and neural activation within the inferior colliculus of guinea pigs, corresponding to the targeted, modelled and then stimulated frequency. There was also no acoustic feedback detected from laser stimulation with our experimental setup. These findings open up the potential for using a convolution-based optoacoustic approach as a new type of laser hearing aid or middle ear implant.

## Introduction

There are about 360 million individuals worldwide who struggle with hearing impairment and have difficulties in communicating on a daily basis^1^. Many of these hearing impaired individuals do not seek help for their hearing loss and can become isolated from society or even lose their jobs. Of the hearing impaired patients who obtain clinical support and receive a hearing aid, a notable proportion of them do not continue to wear and use their hearing devices (up to at least 24%)^2,3^. This lack of use, despite remarkable improvements in the technology of the hearing devices, are in part due to their poor performance or discomfort for a portion of these patients^2,3^. Several factors contribute to the unsatisfactory outcomes of conventional hearing aids, including insufficient frequency specificity or bandwidth, acoustic feedback issues, and discomfort due to the occlusion effect (i.e., earpiece placed into the ear canal to improve sound transmission and minimize acoustic feedback issues when increasing sound amplification) with recurrent auditory canal inflammation^2,3^.

In addition to the conventional hearing aids described above that present acoustic amplification to the ear, there are also hearing devices that directly contact and transmit vibrations to the head or hearing structures^4^. Several types of these devices can be considered as implantable or partial implantable auditory prostheses, including middle ear implants and various bone-anchored hearing devices. The advantages of these implantable hearing devices are that they can transmit greater energy and broader bandwidth of information to the hearing system compared to conventional hearing aids, especially for patients with severe hearing loss who cannot comfortably wear an earpiece that occludes the ear^5^. The disadvantage is that they require surgery for placement with possible complications. For example, passive percutaneous bone conduction hearing devices (skin penetrating and bone anchored) such as the Baha® (Cochlear AG, Sidney, Australia) can have good amplification and sound transmission; however, wound infection around the skin penetrating area^6^ decreases their acceptance in the hearing impaired population. There are also transcutaneous passive bone conduction hearing devices such as the Baha® Attract System (Cochlear AG, Sidney, Australia)^7^ and the Sophono^TM^^8^ or active transcutaneous devices such as the BONEBRIDGE (MED-EL GmbH, Innsbruck, Austria)^9^. However, they have lower sound amplification and modulation capacity due to the transcutaneous interface and can induce pressure issues of the stressed skin between the magnets used to secure the device in its proper location. In cases where bone conduction hearing devices are insufficient, active middle ear implants such as the Vibrant Soundbridge^10^ or Carina ® (Cochlear AG, Sidney, Australia)^11^ provide another option that can lead to satisfying results, but the coupling of the actuator to the middle ear bone structures is challenging in middle ears with pathologies^12^.

Alternative stimulation strategies are therefore needed for the therapy of hearing deficits and pathologies that are still not sufficiently addressed with the current hearing technology. For example, there are patients with chronic inflammation that induces structural changes of the ear drum and middle ear (e.g., cholesteatoma) that can cause chronic leaking and pain within and around the ear as well as damage of structures involved with sound transmission to the inner ear (e.g., ear drum and ossicular chain)^13^. Acoustic hearing aids with an earpiece that occludes the ear could potentially transmit sufficient sound information for these types of patients but occluding the inflamed ear can lead to significant discomfort^5^. Current middle ear implants may not require ear occlusion but cannot be sufficiently attached or adapted to these damaged structures while conventional bone conduction devices are not well tolerated by patients with the tendency for chronic inflammation around the anchor of the bone conduction system^14^. Considering these various limitations experienced by many hearing impaired patients, laser stimulation is emerging as a new form of energy that can be sharply focused to activate specific structures or biological tissue and can be applied without the need for directly contacting the targeted vibratory structures^15–17^. Laser stimulation could be potentially used to vibrate the ear drum with reduced acoustic feedback since the energy transmitted is light instead of sound and without requiring occlusion of the ear canal. If activation of middle ear structures is required due to peripheral ear damage or inflammation, a laser fiber or bundle of fibers could be inserted into the middle ear cavity and directed towards different mechanical structures without requiring attachment to specific locations that may be damaged or inaccessible in some patients, as is encountered for middle ear implants. Therefore, the use of optical energy as a non-contact stimulation method for the activation of the peripheral hearing organ is a promising solution for hearing impaired patients with these types of limitations, and needs to be further explored.

When developing or implementing a device to improve hearing, one needs to keep in mind the fundamental organizing principle of the hearing organ, which exhibits a spatially ordered frequency coding representation across the cochlea (inner ear) that is maintained up through multiple ascending auditory nuclei within the brain^18,19^. Different locations along the cochlea and across neurons within each auditory nuclei are sensitive to specific sound frequencies. The hearing organ and the brain are naturally designed to sense and extract different frequency components of sound over time to encode and elicit intelligible hearing perception. This frequency-specific transmission and extraction of sound first begins through the outer ear canal to the vibratory structures of the middle ear through mechanical vibrations. From the middle ear, the sound pressure waves are transmitted to the inner ear through fluid vibrations within the cochlea that activate inner hair cells along the tonotopically organized cochlea, ultimately reaching the auditory brain through the auditory nerve fibers.

Through the perspective of these physical characteristics of transmission of sound energy from the outer ear to the brain, there are several options for introducing light into the system for hearing purposes: (1) transform the light into mechanical energy (optoacoustic) through very quick laser pulses that lead to mechanical rather than thermal perturbations, in order to directly vibrate outer, middle or inner ear structures^17,20–23^; (2) activate neuronal structures, such as inner hair cells or auditory nerve fibers, directly with light, which is possible with Infrared Neural Stimulation (INS)^15,24^; or (3) transfect the neuronal structures with light sensitive ion channels to become sensitive to laser stimulation, a technique known as optogenetics^5,25,26^. Out of these, INS and optogenetics are appropriate for direct stimulation of the inner ear and the auditory neurons in severe to profound hearing impaired patients. However, the proportion of individuals having conductive, sensorineural or combined hearing loss with residual hearing that can still be activated mechanically is a much larger population, and despite all the improvements in the technology of auditory prostheses, not all patients obtain sufficient or useful hearing. Currently, there are laser-driven hearing devices in which laser is transmitted to a transducer placed at the tympanic membrane (TM),  known as the Earlens device^27^, or the round window^28^ to convert the optical signal to mechanical vibrations in those structures. Both devices demonstrate sufficient vibration amplitudes needed by patients with severe hearing impairment for frequencies up to at least 10 kHz. However, these laser devices require placement of a transducer onto a hearing structure that may not be preferred by some patients or clinicians. The only commercially available laser-based hearing device, the Earlens hearing aid, also requires an intact middle ear.

Therefore, for those individuals not sufficiently benefiting from commercially available hearing devices, a non-contact optoacoustic-based activation approach of the hearing system may offer another hearing option. The first results related to optoacoustic activation of the hearing system was described as artifacts within the inner ear induced through monochrome laser pulses by Friedberger and Ren in 2006^29^. In 2009, Wenzel *et al*. demonstrated that controlled direct optoacoustic stimulation^20,21^ of the inner ear is possible, which was followed up with further experiments demonstrating the ability to directly vibrate structures from the ear drum up to the inner ear without the need for direct contact with the hearing structures^21^.

**Figure 1:**
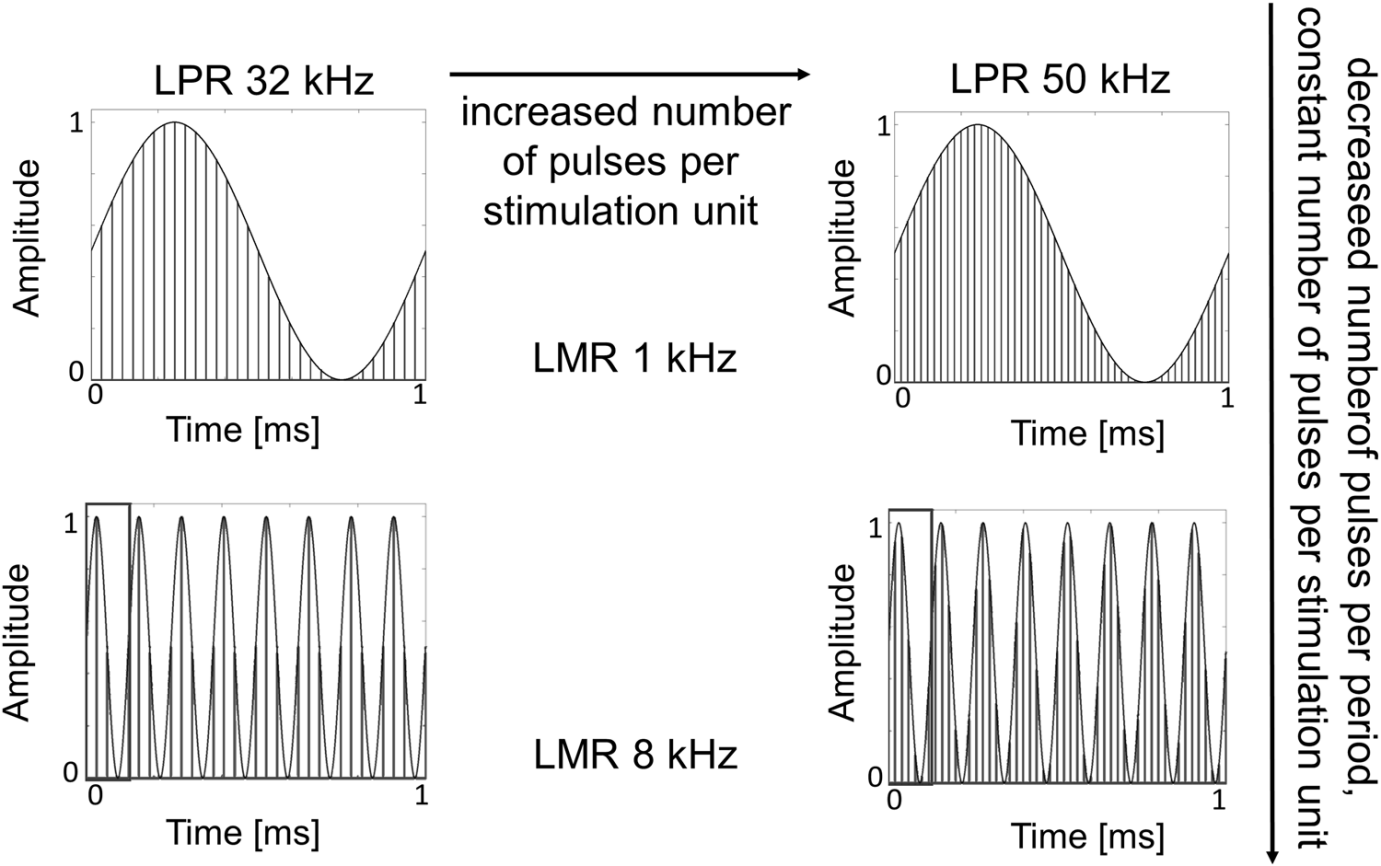
The Optical Pulse Amplitude Modulation. Representation of the optical pulse amplitude modulation paradigm e.g. for 1 kHz and 8 kHz laser modulation rate (LMR). For data comparability we opted to keep the laser pulse rate (LPR) constant over the different modulated frequencies in the presented sets of experiments. As a consequence, the number of pulses and the total energy per sinusoid period varied depending on the LMR as presented in Table 2. Increasing the LMR from 1 kHz to 8 kHz leads to a decreased number of pulses per sinusoid period. Increasing the LPR from 32 kHz to 50 kHz leads to an increased number of pulses per sinusoid and an increased number of pulses per stimulation unit.

There still remain questions on what type of laser pulse patterns to use for optoacoustic stimulation and which peripheral structures to target for sufficient transmission of sound information. For coding speech and other complex signals such as music, one critical question that needs to be addressed based on the fundamental organization of the hearing system described above is if optoacoustic stimulation can induce frequency-specific activation of peripheral hearing structures. This study seeks to answer that question through simulations and experiments in a guinea pig model to demonstrate that laser pulse stimulation of the ear drum and middle ear structures can achieve precise and predictable frequency-specific activation of the auditory system. We were inspired by the amplitude modulation (AM) concept that is well known in radio-frequency engineering, in which a constant carrier frequency could potentially induce vibration waves in the targeted structure that follow the various modulating frequencies (Fig. 1). The activation effects of our laser stimulation paradigm were assessed in three ways: (1) computational modelling with a convolution based model to predict the vibration effects; (2) vibration measurements from the TM performed in extracted peripheral ear specimens in response to laser stimulation; and (3) neural recordings in the central nucleus of the inferior colliculus (ICC) in anaesthetized animals to characterize activation across the well-defined frequency or tonotopic gradient of the ICC in response to laser stimulation. Overall, our results validate the ability to systematically vibrate outer ear and middle ear structures using amplitude modulated laser pulses without requiring contact with the vibratory structures. Frequency-specific activation was also possible with minimal acoustic feedback. Therefore, optoacoustic stimulation offers a valuable tool for contact-free induction of focused mechanical vibrations in targeted biological structures, which can be potentially used for a new generation of hearing aid devices as well as for research purposes.

## Results

### Stimulation principle

**Figure 2:**
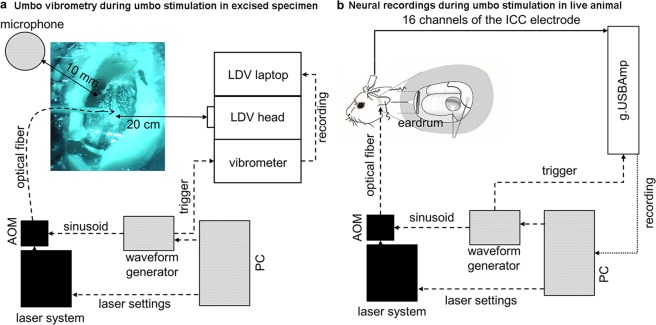
Experimental set up. The trigger-signals for the recordings as well as the sinusoids for the laser stimulation were generated on a PC. They had an onset that was synchronized to laser stimulation. The stimulation laser was operated with a pre-determined laser pulse rate (LPR) of either 32 kHz or 50 kHz. The sinusoid signals were generated with a specific laser modulation rate (LMR) (Fig. 1). The signal (duration of 100 ms with 0.5 ms rise/fall ramp time) was transferred to the input of the acousto-optic modulator (AOM) using the laser fiber (Ø 365 µm) that was connected to the AOM. The distance between the fiber and the tympanic membrane was less than 1 mm. (**a**) For the vibration recordings in response to the laser stimulation in extracted specimens, we placed a scanning laser Doppler (LDV) at a distance of 20 cm from the TM. Using the built-in camera of the LDV, the TM could be displayed on the monitor, the measured points were visualized and the recordings could be monitored and controlled. The acoustic feedback signal was measured at a distance of 1 cm from the ear drum after optical stimulation with 1 kHz and 8 kHz LMR and 50 kHz LPR. (**b**) For the *in vivo* recordings of spike activity in the ICC, a multi-site electrode array with 16 channels was connected via a custom-made head stage to a biosignal amplifier (g.USBamp). The reference was displayed on channel 1 to check the noise level. The raw data was saved for each channel unfiltered for offline analysis on the PC.

We amplitude modulated a predetermined laser pulse rate (LPR, as the carrier frequency) with different laser modulation rates (LMRs) as shown in Fig. 1. In our set of experiments, the LPR was either 32 kHz or 50 kHz, and the LMRs were 1 kHz, 2 kHz, 4 kHz, 8 kHz or 10 kHz, presented individually, to activate audible frequency regions in guinea pigs. Laser Doppler vibrometer (LDV) measurements at the central point of the ear drum (umbo) in explanted specimens (Fig. 2a; see Methods) and neurophysiological recordings in the ICC in anesthetized guinea pigs (Fig. 2b; see Methods) were collected in response to stimulation with these different laser patterns presented to the ear drum or middle ear structures. The averaged maximal power of laser stimulation ranged between 20 and 500 mW, in which even at the highest level causing extensive activity of the auditory system, our calibration microphone still could not detect any laser-induced acoustic energy down to the recording noise floor of 30 dB SPL (Fig. 2a). For our experimental setup, we positioned a microphone (Brüel & Kjær free-field microphone with Type 2670 preamplifier, TYPE 4939-A-011, 2850 Nærum, Denmark) near the ear canal opening and recorded sound signals generated from laser stimulation of the umbo and did not detect any acoustic feedback. Since the form of energy used to stimulate the hearing system is light instead of sound, we expect minimal acoustic feedback. A further explanation could be that our experimental setup was not sensitive or small enough to detect the low sound pressures within the ear canal to assess if there is any acoustic feedback at the optical fiber tip or generated by vibrating structures within the ear canal. This open question will be investigated in future experiments for determining optimal placement of the hearing aid microphone.

### Computational modeling of umbo vibrations

**Figure 3:**
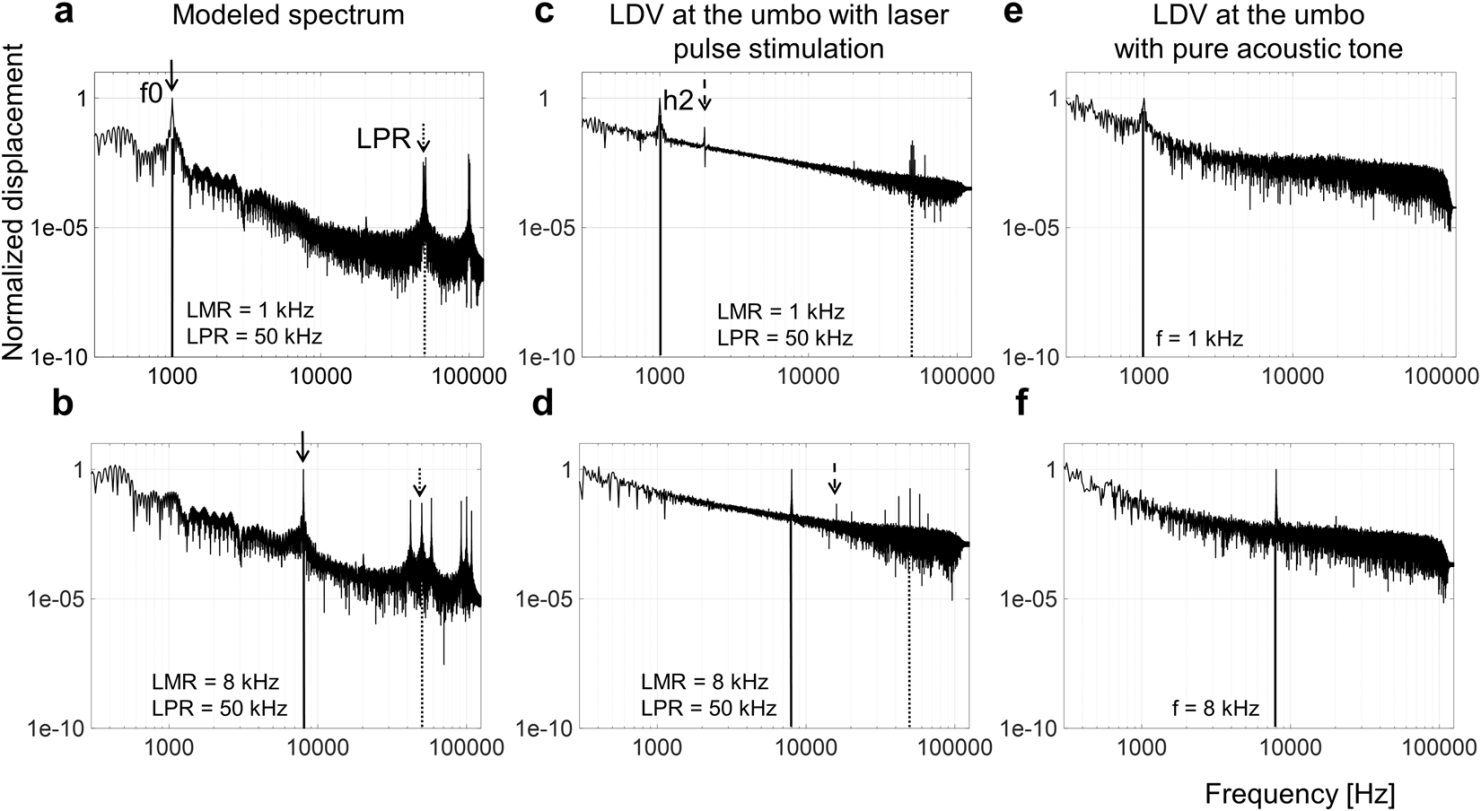
Vibration measurements results. Modeled single side displacement spectra with 50 kHz LPR and 1 kHz (**a**) and 8 kHz LMR (**b**): The peak at the fundamental frequency f0 (solid arrow) could be shifted across the applied LMR from 1 kHz to 8 kHz. An additional peak appeared at the LPR (dotted arrow) with characteristic sidebands, proving our applied modulation method. The normalized single side displacement spectra after optical stimulation with 50 kHz LPR, 1 kHz (**c**) and 8 kHz (**d**) LMR, demonstrate a similar frequency pattern as the recorded displacement spectra. An additional peak appeared at the second harmonic (h2). As a control, normalized displacement spectra after acoustic stimulation with 60 dB SPL acoustic pure tones at the frequencies 1 kHz and 8 kHz are displayed in (**e**) and (**f**), respectively. The fundamental frequency f0 peak is present and consistent with the laser pulse spectra; however, the harmonic components and the LPR peaks caused by the laser pulses are not present.

As the first step of our modeling procedure, we recorded the velocity at the umbo in response to stimulation with a 50 µJ laser pulse (mathematically represented as equation (1)) at the same position. We then calculated the displacement using these velocity data, which is represented as the impulse response of the system: ${\rm{h}}[{\rm{n}}]={\rm{T}}\{{\rm{\delta }}[{\rm{n}}]\}$. The convolution of this impulse response h[n] with an input function representing the desired sequence of laser pulses f[n] (equation (2) led to the model function y[n], which represents the displacement of the umbo vibrations in response to laser stimulation with f[n] (equation (3)) (see Supplementary Fig. S1).

1 $$\,\delta [{\rm{n}}]=\{\begin{array}{c}1\,\,{\rm{for}}\,{\rm{n}}=0\\ 0\,\,{\rm{for}}\,{\rm{n}}\,\ne 0\end{array}$$

2 $$\,{\rm{f}}[{\rm{n}}]=\sum _{{\rm{t}}=-\infty }^{\infty }{\rm{f}}[{\rm{t}}]{\rm{\delta }}[{\rm{n}}-{\rm{t}}])\,{\rm{with}}\,f(t)=\,\sin (2\pi \cdot LMR\cdot t)+1,\,{\rm{with}}\,{\rm{\Delta }}t=\frac{1}{LPR}$$

3 $$\,y[n]=T\{\sum _{\nu =-\infty }^{\infty }f[\nu ]\delta [n-\nu ]\}=\sum _{\nu =-\infty }^{\infty }f[\nu ]T\{\delta [n-\nu ]\}=\sum _{\nu =-\infty }^{\infty }f[\nu ]h[n-\nu ]=\,f[n]\ast h[n]$$

we calculated the single-sided displacement spectrum of y[n] and normalized the data by the value of the first peak (fundamental frequency f0; Fig. 3a,b). As expected, the f0 value consistently aligned with the presented LMR, regardless of the LPR, in which the peak could be shifted to 1 kHz, 2 kHz, 4 kHz, 8 kHz and 10 kHz (1 kHz and 8 kHz are shown in Fig. 3a,b for LPR of 50 kHz; other LMR and LPR examples are shown in Supplementary Fig. S2). The second dominant peak in the modeled spectrum appeared at the LPR as shown in Fig. 3a,b and in Supplementary Fig. S2. The additional frequency peaks in the signal, above and below the LPR peak (i.e., LPR sidebands), confirmed the amplitude modulation characteristic of our stimulation strategy^30^, in which the frequency difference between the LPR peak and the sidebands is equal to the LMR. This could be demonstrated across all the LMR/LPR combinations tested (Supplementary Fig. S2). The harmonic components of the LPR could be identified as well.

### Vibration measurements at the umbo in extracted specimens

**Figure 4:**
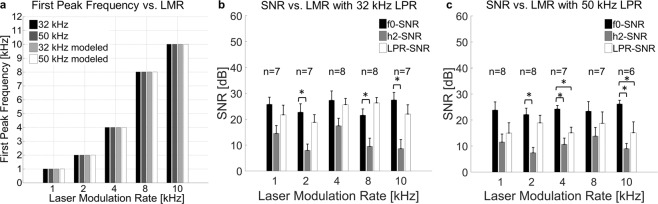
Vibration measurements results – pooled. The mean frequency value of the first peak in each spectrum versus the LMR demonstrates a clear linear mapping for the vibration recordings in in extracted specimens (8 animals) as well as for the modeled data across 7 computations (**a**). The first frequency peak corresponded to the LMR in all cases. We calculated the signal to noise ratio (SNR) at the peaks by dividing the peak frequency (f0, h2, LPR) through the same frequency of the noise signal (i.e., no stimulus condition).The SNR at the LMR peak (black), the h2 peak (grey) and the LPR peak (white) at 32 kHz (**b**) and 50 kHz (**c**) are plotted for comparison in dB (stimulated at 200 mW average peak power). Asterisks correspond to a statistical alpha-level of 0.05, with Bonferroni corrected post-hoc tests.

We validated the modeled frequency specific vibrations at the ear drum level induced through our proposed paradigm by directly recording these vibrations at the umbo with the LDV, in response to stimulation at the umbo, with the actual amplitude modulated laser pulse sequence. These optoacoustic induced vibrations at the umbo were dependent on the applied power as well as the LPR. Using our stimulation paradigm, the fundamental frequency f0 was clearly present in the spectrum and matched the targeted modulation frequency (LMR) (Fig. 3c,d). Consistent with the modeled data, we demonstrated that our novel stimulation paradigm induces the controlled shift of the fundamental frequency f0 from 1 kHz to 2 kHz, 4 kHz, 8 kHz or 10 kHz for both LPRs (Fig. 3c,d are examples for 1 kHz LMR/50 kHz LPR and 8 kHz LMR/50 kHz LPR at an average power maximum of 200 mW; other cases are shown in Fig. S2). The consistency of our results across experiments was analyzed by pooling the modeling data (7 cases) and measured data (8 animals). These data demonstrate that the frequency of the first peak in the spectrum was the same as the applied LMR in all cases (Fig. 4a).

Besides the fundamental frequency f0, the recorded vibration spectra revealed two other main frequency peaks: at the 2nd harmonic (h2 with ${f}_{2}=2\times {{\rm{f}}}_{0}$; Fig. 3c,d) and at the LPR. As reported for the modelled data, additional frequency peaks in the signal above and below the LPR peak (the sidebands) confirmed the amplitude modulation characteristic of our stimulation strategy across all LMR/LPR combinations. As control conditions, we assessed the displacement spectrum of the umbo after acoustic stimulation with pure tones at 60 dB SPL (Fig. 3e,f). These control conditions demonstrated that the fundamental frequency f0 peak in response to optical stimulation is consistent with those elicited by acoustic stimulation, however, without additional h2 and LPR distortion peaks associated with the laser pulses and the modulation paradigm.

The extent of the distortion caused by laser stimulation was further assessed for the different LPR/LMR combinations tested by comparing the signal to noise ratio (SNR) of the fundamental frequency peak (SNR-f0) with the peak at the second harmonic (SNR-h2) and the peak at the LPR (SNR-LPR) (Fig. 4b,c). SNR-f0 was greater than SNR-h2 usually by more than 7 to 20 dB (statistical comparisons across all combinations are presented in Table 1), which may be sufficient to minimize sound distortions produced perceptually by those harmonics. Future psychophysical studies will be needed to confirm the perceptual effects of these distortion components. The SNR-LPR values were typically lower than the SNR-f0 values. The SNR-h2 values were even lower than the SNR-LPR. Additionally, the LPR frequency components are located much higher than the typical audible frequency range for humans and may not be perceptible.

**Table 1:** Statistical Analysis for Fig. 4b,c (asterisks corresponds to p < 0.05).

LPR 32 kHz			LPR 50 kHz		
LMR	Peak	P	Peak	LMR	p
1	F0-H2	0.071	1	F0-H2	0.055
2	F0-H2	0.007*	2	F0-H2	0.001*
4	F0-H2	0.097	4	F0-H2	0.001*
8	F0-H2	0.011*	8	F0-H2	0.281
10	F0-H2	0.006*	10	F0-H2	0.003*
1	F0-LPR	1.000	1	F0-LPR	0.240
2	F0-LPR	1.000	2	F0-LPR	1.000
4	F0-LPR	1.000	4	F0-LPR	0.022*
8	F0-LPR	0.627	8	F0-LPR	1.000
10	F0-LPR	0.824	10	F0-LPR	0.050*

When comparing the variability of the different peaks (f0-SNR, h2-SNR and LPR-SNR), the fundamental frequency f0-SNR also demonstrated the least variation having the highest SNR of 27.4 dB SNR +−1.4 dB SE at 10 kHz LMR/32 kHz LPR and lowest SNR of 21.6 dB SNR +−2.3 dB SE at 8 kHz LMR/32 kHz LPR. The h2-SNR demonstrated higher variability than the fundamental frequency f0 peak across the tested LPR/LMR combinations having the highest SNR of 17.4 dB +−2.9 dB SE at 4 kHz LMR/32 kHz LPR and the lowest SNR of 7.4 dB +−2.1 dB SE at 2 kHz LMR/50 kHz LPR. Additionally, the distortions at the LPR also demonstrated a higher variation than the fundamental frequency f0-SNR having the highest SNR of 26.3 dB +−2.1 dB SE at 8 kHz LMR/32 kHz LPR and the lowest SNR of 15.0 dB +−3.9 dB SE at 1 kHz LMR/50 kHz LPR.

Overall, these results demonstrate the greater detectability and reliability of the fundamental frequency f0 peak compared to the other peaks in the spectrum and the ability to systematically shift the fundamental frequency f0 peak with our modulated laser pulse approach, based on vibration measurements at the umbo.

### Validation of frequency-specific activation measured in the ICC in response to stimulation at the umbo

**Figure 5:**
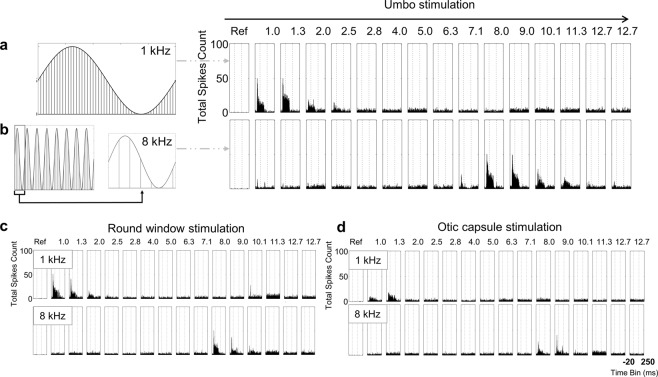
Neural recording results. (**a**) Optical pulse amplitude modulation with 50 kHz LPR and 1 kHz LMR leads to 50 pulses per sinusoid period. (**b**) With a constant LPR of 50 kHz, the chosen paradigm leads to fewer pulses per period for higher LMR in this presented case e.g. 8 kHz. (**a**,**b**) Example of the shift of activity from 1 kHz to 8 kHz *in vivo* after optical stimulation at the umbo (80 mW peak power, 50 kHz LPR). The maximal activity is clearly shifting from one channel to the other in accordance to the LMR value. The optical stimulation at the round window (**c**) led to a similar activation pattern. The optical stimulation at the otic capsule also demonstrated the ability to achieve frequency-specific activation, however it required more energy (e.g., 120 mW) (**d**). The BF of each site is labeled above each set of PSTH plots. PSTHs are based on 100 trials with 1-ms time bins.

To further assess the capabilities of achieving frequency-specific activation of the peripheral hearing system with our developed laser pulse sequences applied at the umbo, the activation effects occurring directly in the auditory brain in a live animal was needed. For this validation, we positioned multi-site electrode arrays within the ICC of anesthetized guinea pigs and recorded neural spiking activity in response to laser pulse stimulation at the umbo using different stimulation parameters. Examples representing post stimulus time histograms, PSTHs, are shown in Fig. 5a,b.

Consistent with the findings from vibration measurements in explanted specimens, optoacoustic stimulation at the umbo with low LMRs elicited activation of the ICC regions most sensitive to low frequencies, whereas optical stimulation with high LMRs elicited activation of the ICC regions most sensitive to high frequencies. We were able to observe a shift of the activated frequency region of the ICC within each animal in accordance to the predicted, calculated and in explanted specimens tested LMR (Fig. 5a,b). The channel with the strongest activation represented the targeted acoustic BF site at threshold corresponding to the LMR.

**Figure 6:**
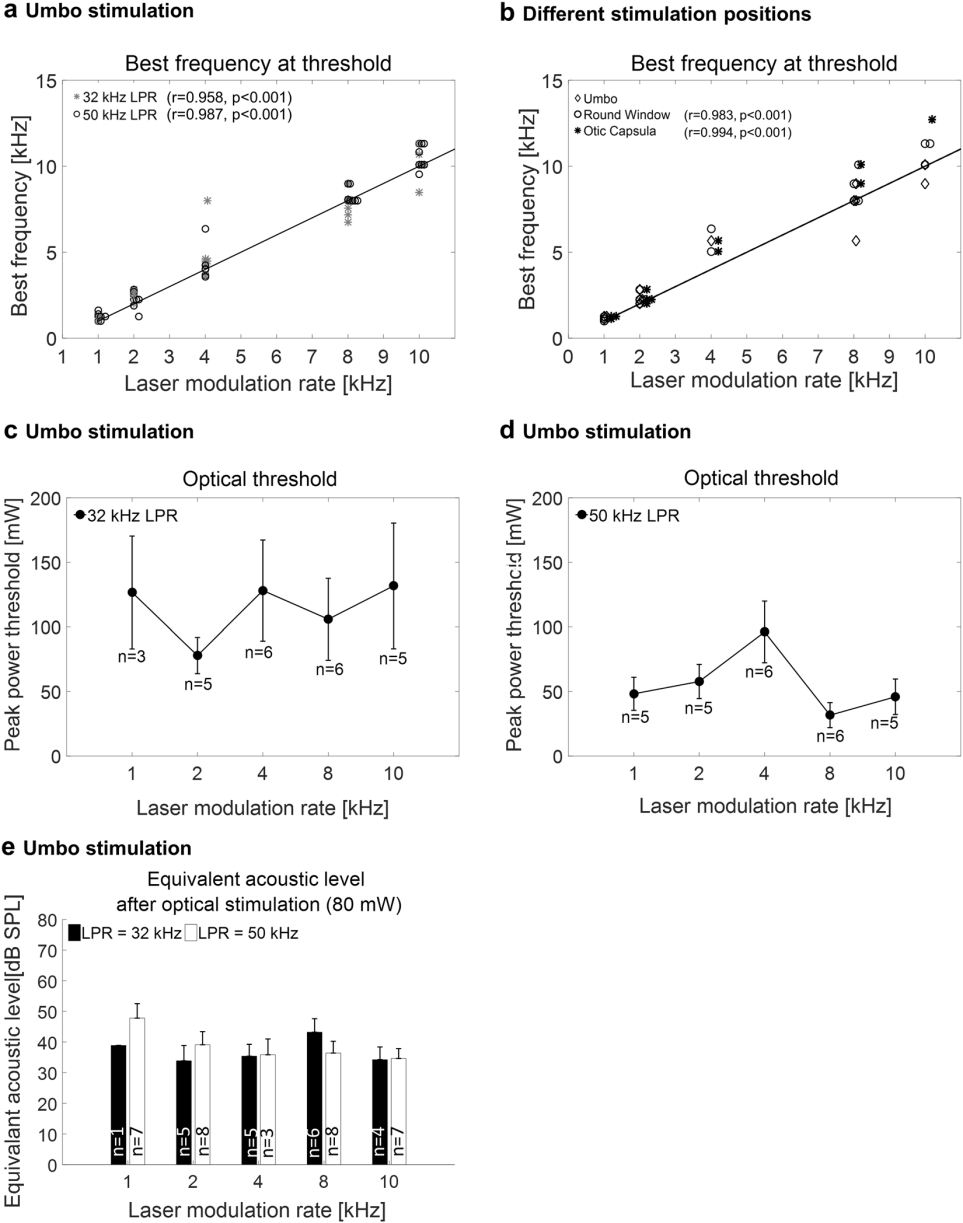
Neural recording results – pooled. (**a**) A linear mapping was demonstrated between the best frequency (BF) at threshold for the most activated site and the LMR in response to optical stimulation at the umbo (32 kHz LPR and 50 kHz LPR). There was strong positive correlation between the two variables LMR and BF at threshold for 32 kHz LPR (p < 0.001, r = 0.958) and 50 kHz LPR (p < 0.001, r = 0.987). (**b**) Comparison of the data recorded at the umbo with the two additional stimulation sites (the round window membrane and the otic capsule) at 50 kHz LPR. The correlation between the two variables LMR and BF at threshold was calculated (p < 0.001, r = 0.983 at the round window membrane and p < 0.001, r = 0.994 at the otic capsule) (n = 4). The optical threshold in peak power at the umbo is displayed for 32 kHz LPR in (**c**) and for 50 kHz LPR in (**d**). The equivalent levels for acoustic stimulation with pure tones in comparison to optical stimulation with 80 mW, using the same frequency as the LMR, is plotted for the different LMR-LPR combinations in (**e**). The data demonstrated mean levels between 34.6 dB SPL (10 kHz LMR, 32 kHz LPR) and 47.8 dB SPL (1 kHz LMR, 50 kHz LPR).

To demonstrate the reliability of our data regarding the frequency-specific activation with optoacoustic stimulation, we analyzed the correlation between the measured BF and the target modulated frequency (LMR) at the optical threshold. Across 12 animals (Fig. 6a), an almost linear mapping from the optical LMR to the acoustic BF of the best activated channel, could be observed. The calculated Pearson correlation analyses for 32 kHz LPR and 50 kHz LPR were significant with a strong positive effect between the BF at threshold and the LMR (p < 0.001, r = 0.958 for 32 kHz; p < 0.001, r = 0.987 for 50 kHz; Fig. 6a).

The optical thresholds were also determined from the PSTH data. Depending on the auditory sensitivity of each animal we observed some variability in the power threshold levels across the tested LMRs, particularly for the 32 kHz LPR (Fig. 6c,d), suggesting that a higher LPR may be more reliable for future implementation. Additionally, our data demonstrated that laser pulse stimulation with 32 kHz LPR (Fig. 6c) required higher power levels than with 50 kHz LPR (Fig. 6d), suggesting the use of higher LPRs may enable safer levels of stimulation in addition to greater reliability of activation (for acoustic control conditions, see Supplementary Fig. S3).

In order to provide an estimate of the acoustic level equivalent for optical stimulation, we analyzed the equivalent acoustic sound pressure level (air conduction) that is comparable to optical stimulation with each LPR-LMR combination at 80 mW (Fig. 6e). The maximal DSR was achieved for 1 kHz LMR and 50 kHz LPR resulting in an acoustic SPL equivalent of 47.8 dB (+−4.7 dB SE).

### Frequency-specific activation for laser stimulation of middle ear structures measured in the ICC

A further advantage of our laser pulse amplitude modulation paradigm is its wide applicability for vibrating different structures in the peripheral auditory system, including the ear drum and structures within the middle ear without needing to be anchored onto or in direct contact with those targeted vibratory structures. Consistent with the results for stimulation at the umbo (ear drum), the applied LMR correlated with the activated BF channels of our ICC electrode for the stimulation at the round window (at the basal end of the cochlea, Fig. 5c) and for stimulation at the otic capsule (the bony encasement of the cochlea, Fig. 5d), in which both locations were accessible in the bulla (middle ear in rodents). Encouragingly, the BF at threshold plotted versus the LMR demonstrated a strong positive linear correlation for stimulation at the round window (p < 0.001, r = 0.983, Fig. 6b-circles) and at the otic capsule (p < 0.001, r = 0.994, Fig. 6b-diamonds), similar to what was observed for stimulation of the umbo (Fig. 6b in same animals, consistent with Fig. 6a from different animals). These results confirm our hypotheses that optoacoustic frequency-specific activation is also possible by presenting laser pulse stimulation to different middle ear structures.

## Discussions

There are about 360 million people worldwide suffering from disabling hearing impairment with at least moderate hearing loss in the better hearing ear, and 32 million of these people are children. This number is rising due to a growing global population and longer life expectancies as well as the increasing noise exposure from the environment, at the workplace and through recreational activities^31^. Although technical and scientific progress has improved the performance level of conventional hearing devices, there are still many hearing impaired people who need alternative hearing solutions. For example, patients with hearing aids struggle in noisy environments and many patients are unable to amplify the sound inputs sufficiently to activate enough frequency bands of information without acoustic feedback distortion, especially those who cannot occlude their ear with an earpiece due to discomfort or inflammation^3^. Middle ear implants and partial implantable bone conduction devices could provide greater amplification for certain hearing-impaired patients without requiring ear occlusion. However, these devices require surgery and anchoring of device components to specific structures. This anchoring as well as the targeted structure can suffer from recurrent inflammation disturbing its vibratory function and/or making device attachment difficult in some patients^12^. Therefore, laser stimulation could serve as a new type of stimulation modality that could potentially enable greater hearing performance across a wider patient population by offering a non-contact vibratory input with minimal or no acoustic feedback issues.

The first non-ablative therapeutic use of laser into the inner ear, the cochlea, was reported in 2004^32^ with the purpose to modulate cochlear mechanics. Infrared laser light as a stimulation method for peripheral nerve activation (INS)^15,24^ as well as the optogenetic stimulation approach^16,26^ have been reported and target patients with severe to profound sensorineural hearing loss (i.e., significant loss of functional hair cells). Additionally to INS, the “optophonic” stimulation of the inner ear with near infrared laser light has been observed^23,33^ in which sound could be generated by the laser stimulus that vibrated and activated intact auditory sensory cells (hair cells). Another laser approach described by Wenzel *et al*.^17^ demonstrated the activation of the inner ear with green pulsed laser light (532 nm) using the optoacoustic effect by delivering very short pulses that induced direct vibrations of the cochlear basilar membrane. In the current study, we have extended this previous work by demonstrating that optoacoustic stimulation can also vibrate structures within the outer and middle ear without requiring direct contact with those targeted structures. By presenting amplitude modulated pulse patterns using a convolution-based method, optoacoustic stimulation can achieve controllable frequency specific activation at the ear drum and more centrally in the ICC, which is a critical requirement for a hearing device.

One advantage over other laser-based hearing devices designed for the outer or middle ear, such as the Earlens, is that the optoacoustic approach would not require a sensor-actuator component be attached to the hearing system (e.g., the ear drum). Similar to the Earlens^27,34^, since the transmitted energy is light instead of sound, acoustic feedback can be greatly reduced for an optoacoustic-based hearing aid, potentially providing greater frequency bandwidth and sound quality to the patients without requiring full occlusion of the ear canal. In cases where there is damage to certain regions of the middle ear pathway (e.g, ossicular chain and/or the round window niche being not sufficiently accessible), in which the Earlens or conventional middle ear implants may not be suitable, an optoacoustic hearing device could be implanted into the middle ear cavity with a fiber or fiber bundle targeting bone regions beyond the damaged area, especially in cases where there is no reliable location to attach the actuator of a middle ear implant.

There are still several challenges that need to be addressed before an optoacoustic hearing device can be translated into patients. First, an optical fiber or fiber bundle needs to be positioned safely and stably into the ear canal or into the middle ear cavity. One option for the outer ear canal would be to embed the fiber(s) into a vented earpiece that can be inserted into the ear canal or in the inner portions of the concha. The location of the microphone should be positioned as close to the entrance of the ear canal to take advantage of the high-frequency pinna-diffraction, possibly embedding it in the same earpiece, assuming acoustic feedback within the ear canal caused by the vibrating ear drum is minimal^34^ and needs to be characterized in a future study. Within the middle ear cavity, the fiber(s) could be embedded in a miniaturized and biocompatible guide tube or casing that is fixed to healthy bony wall within the mastoid and the fiber(s) can be oriented towards different portions of the ossicular chain or bony area in close proximity to the inner ear, round window or surrounding bony structures (e.g. promontorium).

Second, a stimulation strategy is needed that can transmit complex signal patterns, such as speech and music, to the outer and middle ear structures. The fact that we were able to reliably predict the measured vibratory response at the ear drum level using a LTI modelling approach (i.e., based on a linear, time-invariant assumption) provides optimism that the results observed in our study with simple pure tones and sinusoid modulated laser pulse sequences may reasonably translate to more complex inputs, in which those complex signals can be modelled as a sum of pure tones in a LTI framework. The brain is undoubtedly a non-linear system, and thus further experiments will still need to be pursued to assess how the responses in the ICC and auditory cortex, as well as hearing perception, actually compares between the complex acoustic inputs and the laser pulse sequences modulated by those complex inputs (e.g., by the envelope and/or fine structure features of those inputs). Third, we need to determine the safe range of levels of optoacoustic stimulation within the outer and middle ear and if the maximum level is sufficient to provide a wide dynamic range for useful hearing. Finally, we need to calculate the total energy required to drive an optoacoustic hearing device and to minimize overall consumption to ensure the device can last at least for a full day with daily charging of the device overnight.

In summary, we have developed and evaluated a laser-based pulse amplitude modulation approach that can provide a versatile and non-contact method to precisely vibrate structures within the peripheral hearing system. This new type of technology could provide an alternative solution for implantable and non-implantable hearing aids by replacing the speaker or the sound transducer (e.g., force mass transducer in a middle ear implant) with a non-contact and focused energy transfer modality via laser pulses to be used in hearing impaired patients who are not sufficiently satisfied or aided with current hearing aid technologies.

## Material and Methods

### Animal model

We chose albino guinea pigs (Charles River Laboratories, Sulzfeld, Germany) according to the protocols and the procedures approved by the Animal Welfare Office of the University of Saarland and by the Central Veterinary Office of Saarland (TV27/2011; TV41/2015). We present data from 27 animals, in which 9 animals were used for the umbo vibration measurements in explanted specimens and 18 animals were used for the neural recordings in the inferior colliculus. The guinea pigs weighing 350–900 g were anesthetized with 40 mg/kg ketamine (Ketanest, Albrecht, Aulendorf/Württemberg, Germany) and 10 mg/kg xylazine (Rompun, Bayer Health Care, Leverkusen, Germany) applied intra muscular. For the LDV experiments in extracted specimens the animals were sacrificed in deep anesthesia. For the neural recording experiments the animals were kept anesthetized throughout the experiment.

### Acoustic stimuli and trigger signals

We generated the acoustic- and the trigger-signals on a PC (Hewlett-Packard Company /HP Inc., Palo Alto, CA, USA) in the control room (Fig. 2). We transferred the MATLAB® (R2014a, The MathWorks Inc., Natick, USA) generated sinusoid (duration of 100 ms with 0.5 ms rise/fall ramp time) to a waveform generator (33500b Waveform Generator, Agilent Technologies, Santa Clara, USA) as an arbitrary file via Virtual Instrument Software Architecture (VISA) interface. We regulated the sound pressure level through a programmable attenuator (g.PAH, g.tec medical engineering GmbH, Schiedlberg, Austria) and transmitted the acoustic signal through a free field loudspeaker. The speaker was positioned and calibrated for a 10-cm distance to the left ear. The trigger signal for the recordings lasted 100 µs and had an onset that was synchronized to the laser or acoustic stimulation signal.

### Laser

We used a 532 nm pulsed Neodymium-doped Yttrium Orthovanadate (Nd:YVO4) laser system (INCA, Xiton Photonics GmbH, Kaiserslautern, Germany). The stimulation laser was operated with a pre-determined laser pulse rate (LPR) of either 32 kHz or 50 kHz. We generated the sinusoid signal with a specific laser modulation rate (LMR) (Fig. 1) as described for the acoustic signals. The signal (duration of 100 ms with 0.5 ms rise/fall ramp time) was transferred to the input of the acousto-optic modulator (AOM) (Xiton Photonics GmbH; Fig. 2a). The laser pulses were then delivered to the target structure (ear drum, otic capsule, round window) using the laser fiber (Ø 365 µm) that was connected to the AOM.

To keep the number of pulses per stimulation unit constant over the different modulated frequencies tested, the number of pulses under the envelope had to vary from one modulated frequency to the other as presented in Table 2.

**Table 2:** Calculations for the number of pulses per sinusoid period for different LPR/LMR combinations.

LPR in kHz	LMR in kHz	No of pulses	LPR in kHz	LMR in kHz	No of pulses
32	1	32	50	1	50
32	2	16	50	2	25
32	4	8	50	4	12.5
32	8	4	50	8	6.25
32	10	3.2	50	10	5
32	16	2	50	16	3.125

We changed the amplitude of the laser pulses corresponding to the LMR at 1 kHz, 2 kHz, 4 kHz, 8 kHz and 10 kHz from an averaged maximal peak power between 20 and 500 mW (Table 3).

**Table 3:** Relationship between Measured Peak Power [mW] and Energy per pulse [µJ] for different LPR.

LPR in kHz		Measured Peak Power [mW]	Energy/pulse [µJ]	LPR in kHz	Measured Peak Power [mW]	Energy/pulse [µJ]
				50	20	0.4
32		32	1	50	30	0.6
32		48	1.5	50	50	1
32		83.2	2.6	50	75	1.5
32		128	4	50	130	2.6
32		201.6	6.3	50	200	4
32		320	10	50	315	6.3

### Statistical analysis

The data is presented with line plots or bar plots displaying the mean values and standard error of the mean calculated with MATLAB®. The Statistical Analysis was performed with SPSS (IBM Corp. Released 2013. IBM SPSS Statistics for Windows, Version 22.0. Armonk, NY: IBM Corp.). For the *vibration* measurements in extracted specimens in Fig. 4b,c, we conducted one factorial ANOVAs after checking the data regarding the normal distribution and variance homogeneity. For the comparison between the individual groups, we performed post-hoc Bonferroni tests. The reported alpha level was 0.05.

### Vibration measurements in explanted specimens

#### Surgical technique and laser Doppler vibrometer setup

We explanted the temporal bones, removed the cartilaginous outer ear canal and exposed the tympanic membrane (TM). To improve the signal to noise ratio (SNR), we placed micron sized glass beads (Ø 50 µm/bead) on the TM. With a custom-made holder we fixed the head, inserted the laser fiber into the outer ear canal and directed it towards the TM. For the vibration recordings in response to the laser stimulation, we placed a scanning laser Doppler vibrometer PSV 500 (Polytec GmbH, Waldbronn, Germany) (LDV) at a distance of 20 cm from the TM. Using the built-in camera of the LDV, the TM could be displayed on the monitor, the measured points were visualized and the recordings could be monitored and controlled (Fig. 2a). In order to obtain an adequate SNR, the scan points had to be set exactly on the reflective glass beads.

### Stimuli and procedure

#### Stimuli

For the acoustic control measurements at the beginning of the LDV recordings in each experiment we applied pure tones (1 kHz, 2 kHz, 4 kHz, 8 kHz and 10 kHz) with 0 dB SPL, 60 dB SPL, 70 dB SPL and 80 dB SPL. (Supplementary Fig. S4). These data enabled comparisons between the acoustic- and the optical-induced vibrations of the ear drum. The averaged maximal power for laser stimulation was between 20 and 500 mW (Table 3). The acoustic and optical stimulation rate was 5 stimulation units/s. A minimum of 17 runs were recorded per frequency/level combination and the results were analyzed offline.

#### Measurements

We connected the vibrometer with the external trigger signal of the waveform generator (33500b Waveform Generator, Agilent Technologies, Santa Clara, USA) and with the laser scanning head that was connected to the laptop running the Polytec GmbH control software (Fig. 2a). We performed the velocity measurements in the time domain and saved these for offline analysis with Polytec File Access Software in combination with MATLAB®. After the 2D-adjustment, the scan points were set.

### Data analysis

#### Offline processing

To suppress artifacts, we used a bandpass filter between 300 and 12000 Hz and averaged a minimum of 17 runs. We calculated the displacement from the measured velocity data through numerical integration, and determined the single sided amplitude spectra using Fourier transformation. The fundamental frequency as well as the distortions were analyzed.

#### Signal to noise ratio (SNR)

We calculated the SNR at the peaks by dividing the peak frequency (f0, h2, LPR) by the same frequency of the noise signal (i.e., no stimulus condition). We then converted the SNR to a dB scale using equation (4). We discarded measurements if the f0-SNR was below 6 dB. If the h2-SNR or the LPR-SNR was below 0 dB it was set to 0 dB.

4 $${SNR}=20\times \,\mathrm{log}(\frac{pea{k}_{signal}}{pea{k}_{noise}})$$

### Convolution based computational modeling

As described in signal processing theory^35^, the output of a linear time invariant (LTI) system can be calculated by convoluting the impulse response of the system with an input signal. Based on this idea, we implemented a convolution based model in MATLAB®: We recorded the LDV vibration velocity at the umbo in response to optical stimulation with a 50 µJ laser pulse, which is as an approximation to the Dirac impulse (equation (1)).

We considered the LDV velocity recording in response to stimulation with a 50 µJ laser pulse that can be represented as the impulse response of the system using this equation: ${\rm{h}}[{\rm{n}}]={\rm{T}}\{{\rm{\delta }}[{\rm{n}}]\}$. The convolution of these impulse response h[n] with an input function representing the laser pulses (equation (2)) leads to the model function y[n] (equation (3)). We compared this modeled function with the displacement measurements made at the umbo in response to the actual laser modulated pulse stimulation (Supplementary Fig. S1).

### Neural recordings in the ICC in anesthetized animals

#### Animal model, anesthesia and surgical procedure

For the neural recordings in animals, we extended the initial anesthesia described above with additional 1/4–1/2 of the initial dosage every 30 to 60 minutes to maintain an areflexive state. We injected 50 ml/kg bodyweight saline subcutaneous per day divided in dosages every 1–2 hours. The body temperature was stabilized at 38 °C during the experiment using a DC heating pad. We removed the left auricle and exposed the meatus acusticus externus to attain access to the TM. Additionally, through a retoauricular incision we opened the middle ear (the bulla) and exposed the cochlea. We fixed the head with a custom-made holder, opened the skull and exposed the inferior colliculus. We inserted an A1x16 single shank electrode (NeuroNexus Technologies, Ann Arbor, MI, USA) in an angle of 45° to the horizontal line using a micromanipulator (MN-151, Narishige International Limited, London, UK) with a custom built angle adjusting system in order to position the shank along the tonotopic gradient of the ICC^36,37^. We placed platinum subdermal needles as a reference signal (vertex) and ground (neck). We then performed the acoustic stimulation to determine the final position of the electrode (for details see *Stimuli and recordings*) and covered the brain with agar to reduce its swelling, pulsations and drying. We inserted the laser fiber into the outer ear canal and directed it towards the TM using a second micromanipulator (Mk1 Manipulator, Singer Instruments, Roadwater, UK) (n = 12 animals). For other stimulation locations, we directed the fiber towards the round window membrane or the otic capsule at the basal turn level (n = 4 animals). The LPR was 50 kHz in those set of experiments.

#### Acoustic and optical stimuli and recordings

The A1x16 electrode had an inter channel spacing of 100 µm and an area of 703 µm^2^ and was connected via a custom-made head stage to the biosignal amplifier (g.USBamp, g.tec medical engineering GmbH, Schiedlberg, Austria) (Fig. 2b). The reference was displayed in channel 1 to check the noise level. The recording software was implemented in Simulink® (The MathWorks Inc., Natick, USA). We saved the raw data for each channel unfiltered for offline analysis. To check for the correct insertion depth and position inside the ICC, we performed online analysis (local field potentials (LFP) for each channel, spike activity and total spike rate (TSR)) in response to acoustic stimulation with pure tones (1 kHz, 2 kHz, 4 kHz, 8 kHz and 10 kHz) at different levels (40–60 dB SPL in 10 dB steps). The stimulation rate for all acoustic and optical stimulation sets *in vivo* was 1.86 stimulation units/s. The spike activity was bandpass filtered between 300 and 3000 Hz as described by Lim *et al*.^38^ and the spike trains were displayed 10 ms before and 250 ms after the stimulation trigger signal for each channel. We calculated the LFP by averaging the neural signal for each channel over 20 trials after filtering between 20 and 3000 Hz. We calculated the TSR by dividing the number of spikes in the 5 to 105 ms interval after the trigger onset by 100 ms. For 3 animals, each level-rate combination of our optical stimulation paradigm was performed twice, in which the second time was performed in reverse ordering (backwards) from the first one, to prove the reliability of our recordings.

### Data Analysis

We performed the data analysis in response to the acoustic and the optical stimulation using a spike detection algorithm. Spikes were defined as signal peaks having values with a standard deviation of 3.5 above the background noise. The time at which the spike was detected (timestamp) corresponded to the largest negative peak of that spike (Supplementary Fig. S5).

#### Best frequency (BF) and driven spike rate (DSR)

To determine the BF for each electrode channel, we applied acoustic pure tone stimulation in a random order for six frequencies per octave between 2 kHz and 32 kHz (high frequency electrode position) or between 1 and 22 kHz (low frequency electrode position) depending on the probe position of each experiment (n = 5 animals for high frequency experiments, n = 11 animals low frequency experiments). We generated the acoustic frequency response map (FRM) online with at least 7 runs per frequency and level pair (Supplementary Fig. S6a).

To determine the DSR for a set time window, we subtracted the spontaneous spike rate (SSR) from the TSR for each channel. We then normalized the DSR by the maximum spike rate per channel across the presented stimuli and plotted the normalized spike rates as a FRM (Supplementary Fig. S6a). We determined the BF as the frequency with the maximum activation at 10 dB SPL above the visually-determined acoustic threshold per channel. The BF versus site number plot demonstrated the tonotopy of our insertion (Supplementary Fig. S6b).

#### Post Stimulus Time Histograms (PSTHs)

We used PSTHs with timestamps across at least 100 runs (1-ms bin) to analyze the data and determine the neural response on each channel after acoustic or optical stimulation. The corresponding acoustic BFs were mapped to each histogram channel for further assessment (Supplementary Fig. S7).

#### Equivalent Level Estimation between Acoustic and Optical Stimulation

For the estimation of the acoustic sound pressure level equivalent for pure tone stimulation in comparison to optical stimulation, the ICC spike activity in response to optical stimulation with 80 mW for each LPR-LMR combination was recorded. The DSR was compared to the acoustic level at the same electrode channel after stimulation with the best frequency. We discarded values, if the BF channel at maximal acoustic stimulation differed more than 1 channel from the BF channel at threshold.

### Code availability

Custom code for recording and analysis is available from the corresponding authors on reasonable request.

## Supplementary information

Supplementary Figures

## Data Availability

The datasets generated during and/or analyzed during the current study are available from the corresponding authors on reasonable request.
