# Supplementary material for: Frequency-specific activation of the peripheral auditory system using optoacoustic laser stimulation
Source: Sci Rep. 2019 Mar 12;9:4171. doi: 10.1038/s41598-019-40860-8 (PMC6414650; doi:10.1038/s41598-019-40860-8)
Supplement: Supplementary file 1 — Supplementary Figures [file 41598_2019_40860_MOESM1_ESM.pdf]

Supplementary figures for:

Frequency-specific activation of the peripheral auditory system using optoacoustic laser stimulation

Patricia Stahn[1a)*], Hubert H. Lim[2], Marius P. Hinsberger[1], Katharina Sorg[1], Lukas Pillong[1], Marc Kannengießer[1,4], Cathleen Schreiter[1], Hans-Jochen Foth[3], Achim Langenbucher[4], Bernhard Schick[1], Gentiana I. Wenzel[1b)*]

[1]Saarland University, Faculty of Medicine, Department of Otolaryngology,
Kirrbergerstr. 100, 66421 Homburg, Germany.

[2]University of Minnesota, Department of Biomedical Engineering, Department of Otolaryngology

[3]University of Kaiserslautern, Department of Physics

[4]Saarland University, Experimental Ophthalmology

[a)] Corresponding author:

Patricia Stahn

Department of Otolaryngology, Saarland University

Kirrbergerstr. 100

D-66421 Homburg/Saar

Germany

Tel.: +49 (0) 6841/1612902

E-Mail: patricia.stahn@uks.eu

[b)]

PD Dr. med. Gentiana I. Wenzel

Department of Otolaryngology, Saarland University

Kirrbergerstr. 100

D-66421 Homburg/Saar

Germany

Tel.: +49 (0) 6841/1612903

E-Mail: gentiana.wenzel@uks.eu

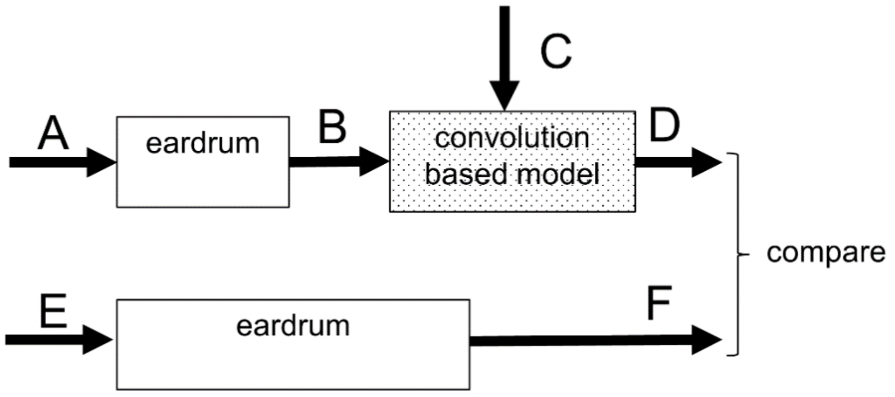

Supplementary Figure S1. Outline of the convolution based modelling of the tympanic membrane vibrations. The single pulse applied at the eardrum (A) is recorded with the LDV (B). The model input function (C) is convoluted with the recorded displacement impulse response (B). The resulting model output function (D) is compared with the direct LDV recordings at the eardrum (F) after the application of the same laser pulse pattern (E). We analyzed (D) and compared it with the modelled displacement spectrum (F).

(a)

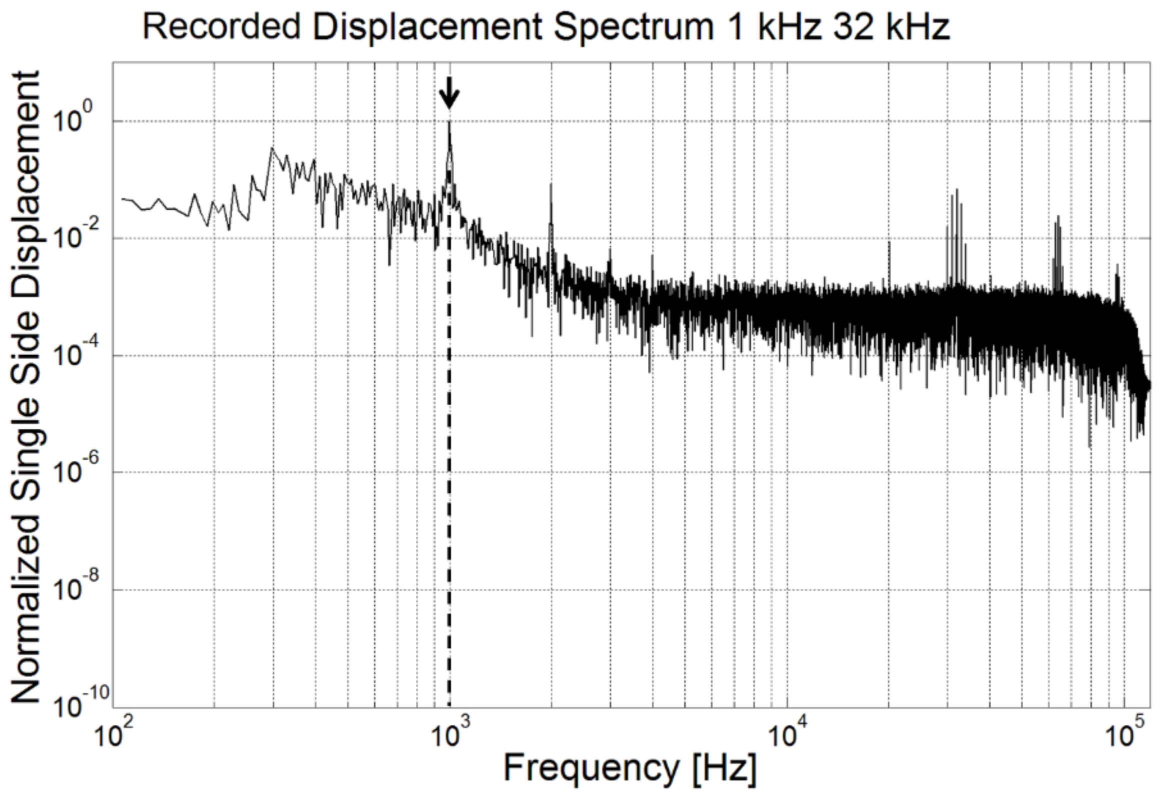
Recorded Displacement Spectrum 1 kHz 32 kHz
Normalized Single Side Displacement
10^0
10^-2
10^-4
10^-6
10^-8
10^-10
10^2
10^3
10^4
10^5
Frequency [Hz]

(b)

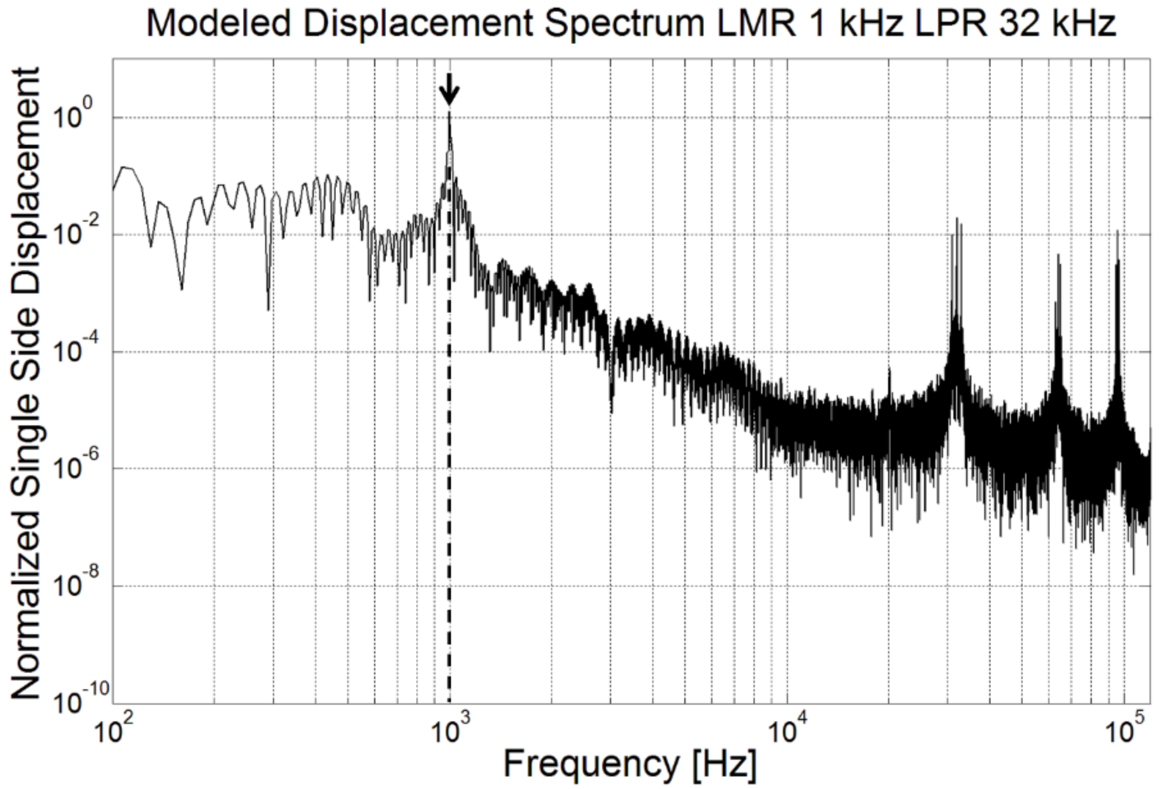
Modeled Displacement Spectrum LMR 1 kHz LPR 32 kHz
Normalized Single Side Displacement
10^0
10^-2
10^-4
10^-6
10^-8
10^-10
10^2
10^3
10^4
10^5
Frequency [Hz]

(c)

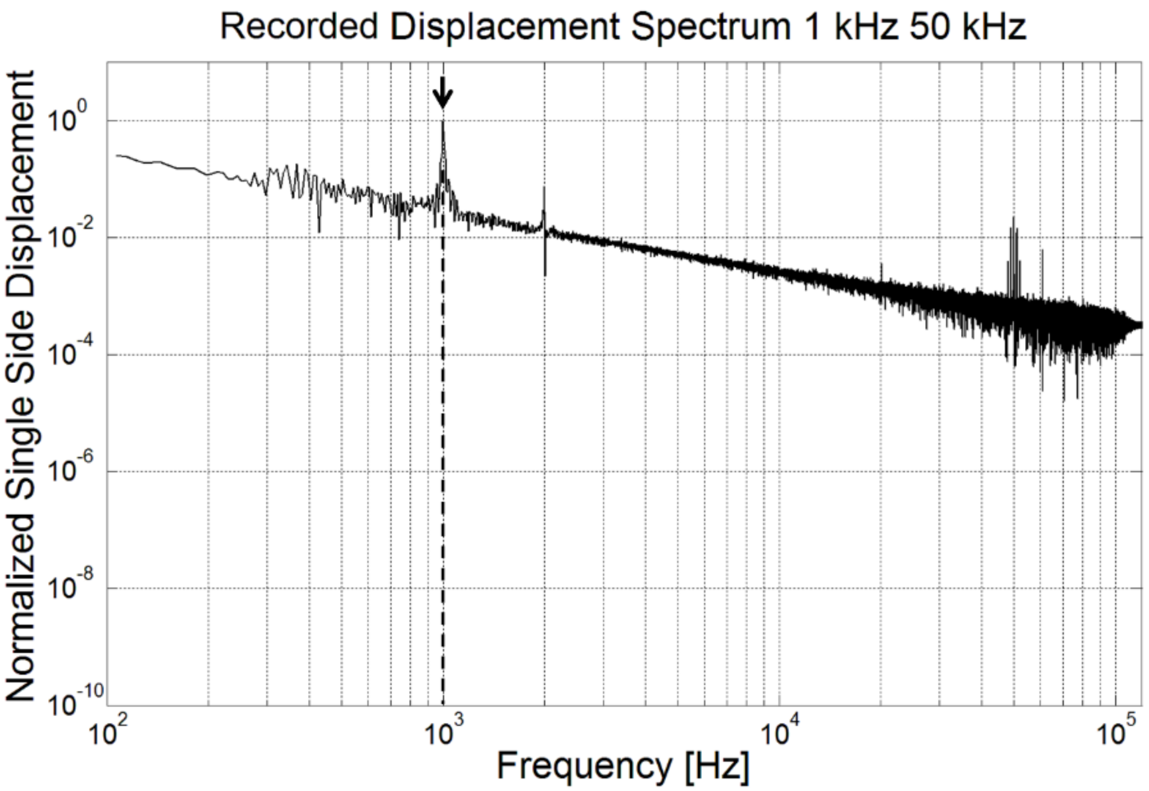
Recorded Displacement Spectrum 1 kHz 50 kHz
Normalized Single Side Displacement
10^0
10^-2
10^-4
10^-6
10^-8
10^-10
10^2
10^3
10^4
10^5
Frequency [Hz]

(d)

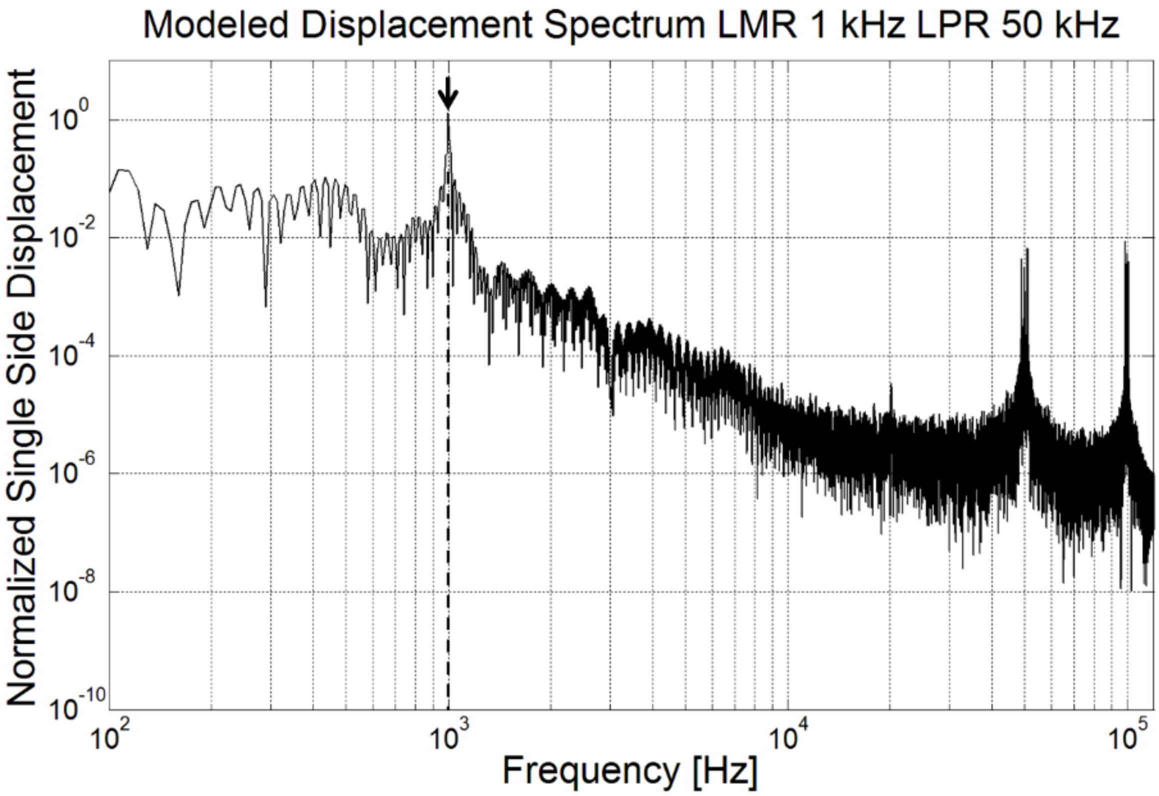
Modeled Displacement Spectrum LMR 1 kHz LPR 50 kHz
Normalized Single Side Displacement
10^0
10^-2
10^-4
10^-6
10^-8
10^-10
10^2
10^3
10^4
10^5
Frequency [Hz]

(e)

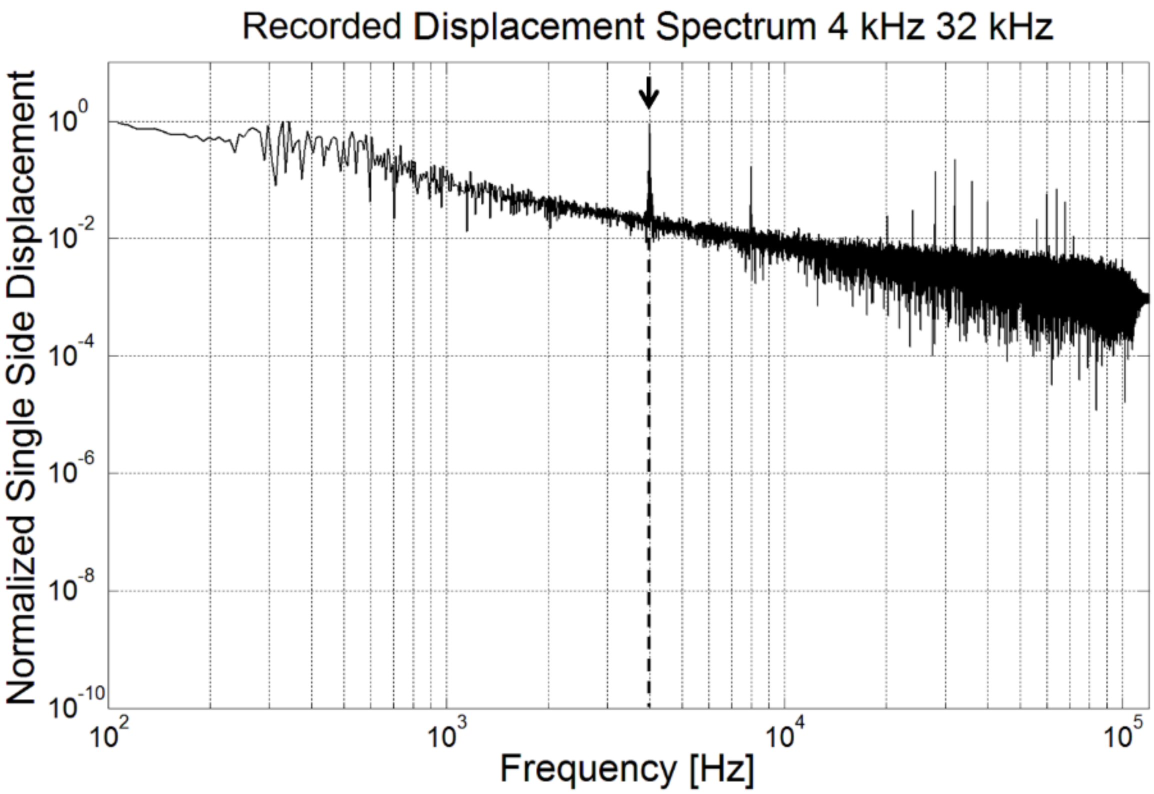
Recorded Displacement Spectrum 4 kHz 32 kHz
Normalized Single Side Displacement
10^0
10^-2
10^-4
10^-6
10^-8
10^-10
10^2
10^3
10^4
10^5
Frequency [Hz]

(f)

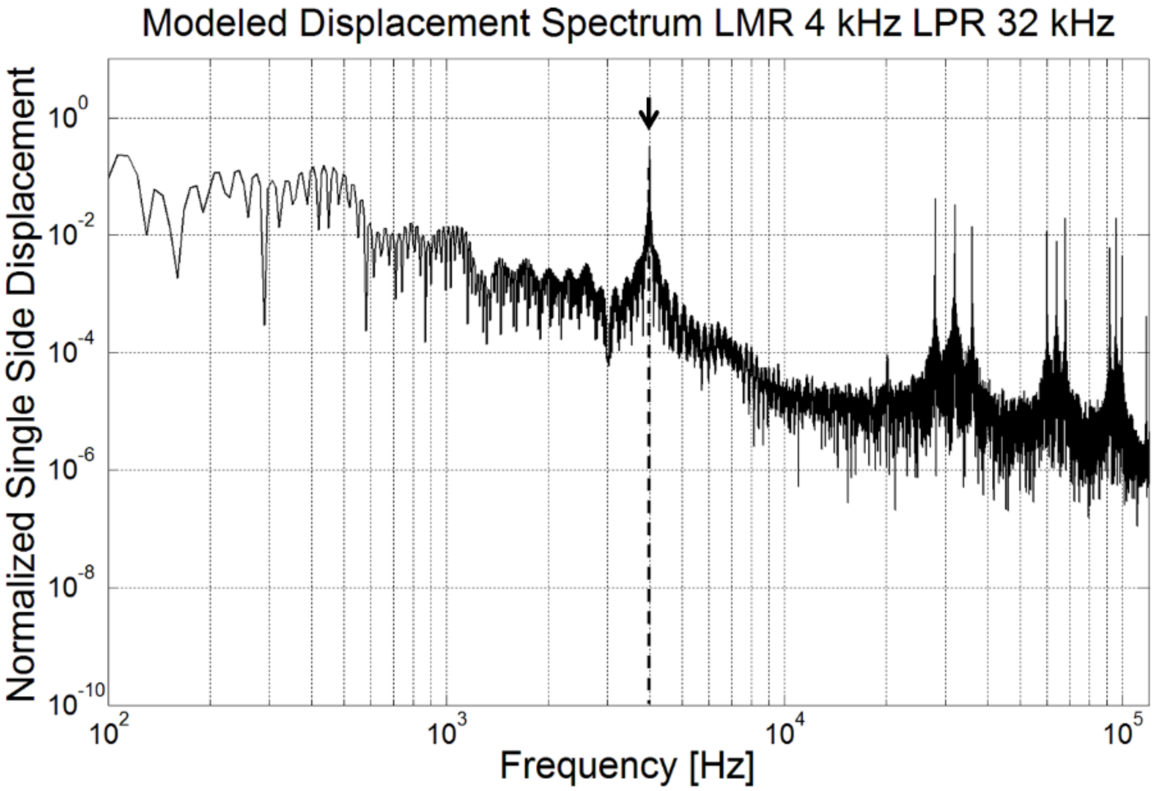
Modeled Displacement Spectrum LMR 4 kHz LPR 32 kHz
Normalized Single Side Displacement
10^0
10^-2
10^-4
10^-6
10^-8
10^-10
10^2
10^3
10^4
10^5
Frequency [Hz]

(g)

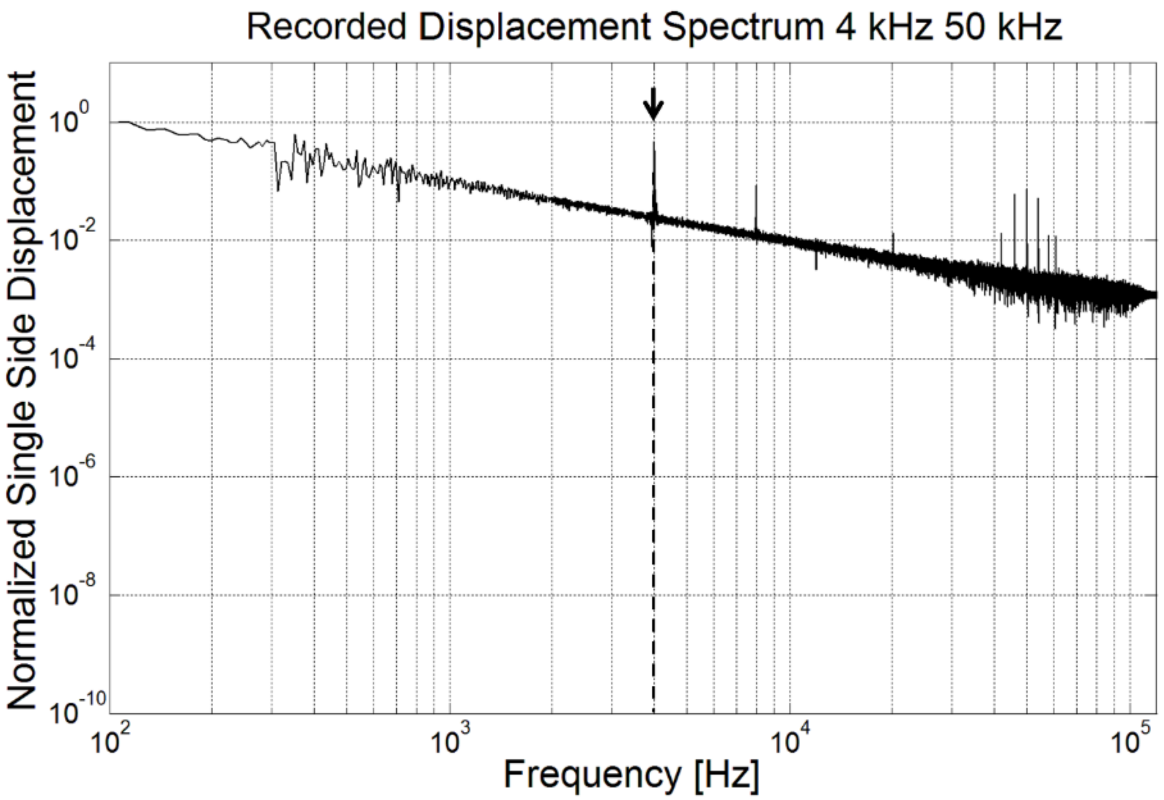
Recorded Displacement Spectrum 4 kHz 50 kHz
Normalized Single Side Displacement
10^{0}
10^{-2}
10^{-4}
10^{-6}
10^{-8}
10^{-10}
10^{2}
10^{3}
10^{4}
10^{5}
Frequency [Hz]

(h)

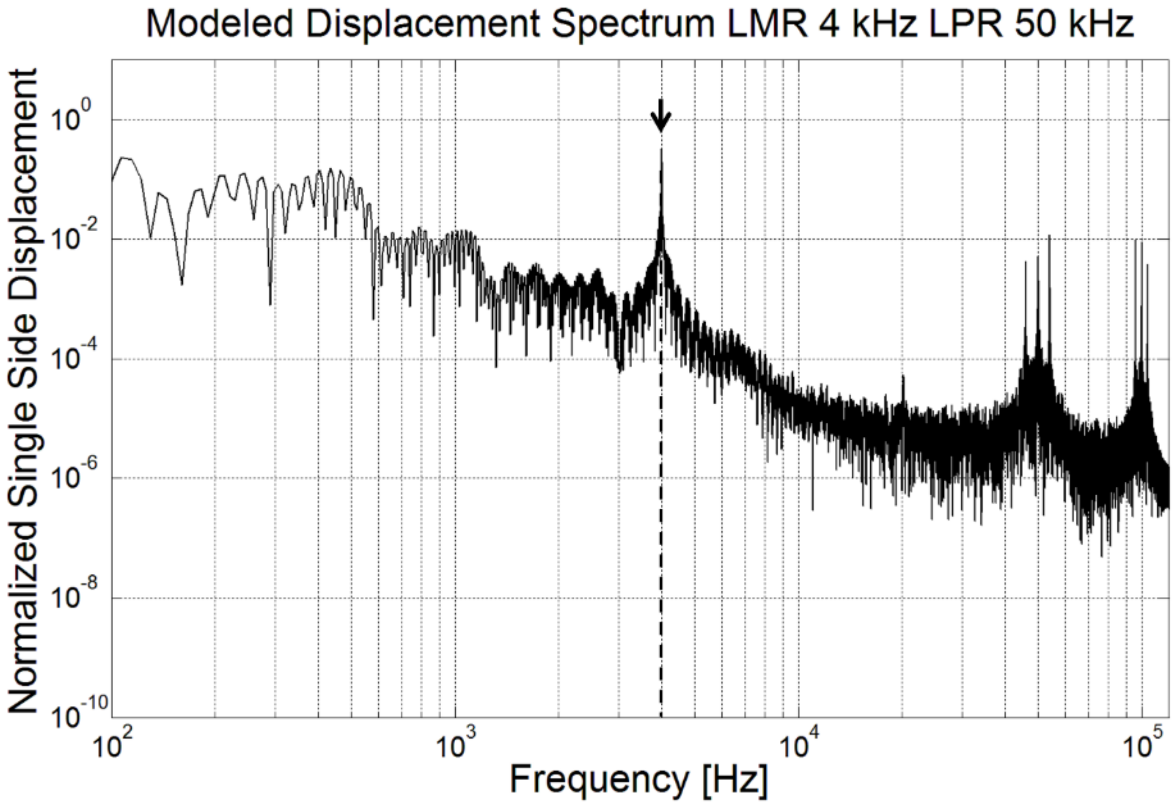
Modeled Displacement Spectrum LMR 4 kHz LPR 50 kHz
Normalized Single Side Displacement
10^{0}
10^{-2}
10^{-4}
10^{-6}
10^{-8}
10^{-10}
10^{2}
10^{3}
10^{4}
10^{5}
Frequency [Hz]

(i)

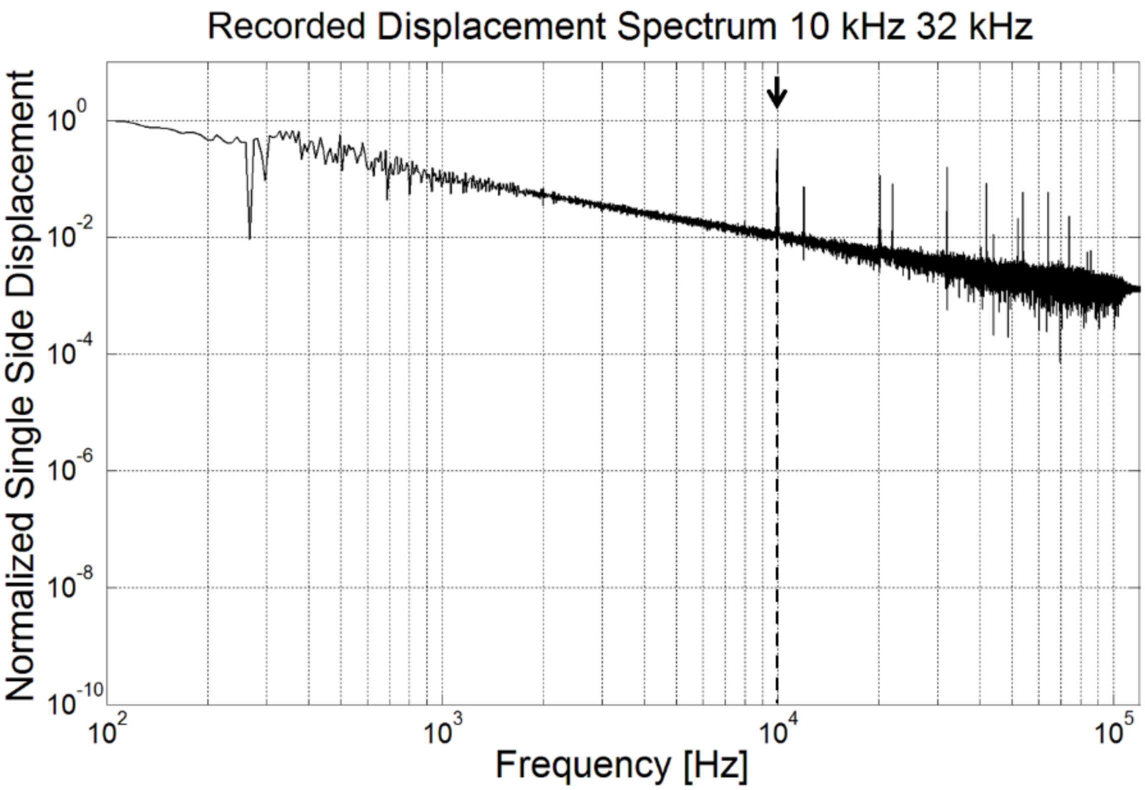

Recorded Displacement Spectrum 10 kHz 32 kHz
Normalized Single Side Displacement
10^0
10^-2
10^-4
10^-6
10^-8
10^-10
10^2
10^3
10^4
10^5
Frequency [Hz]

(j)

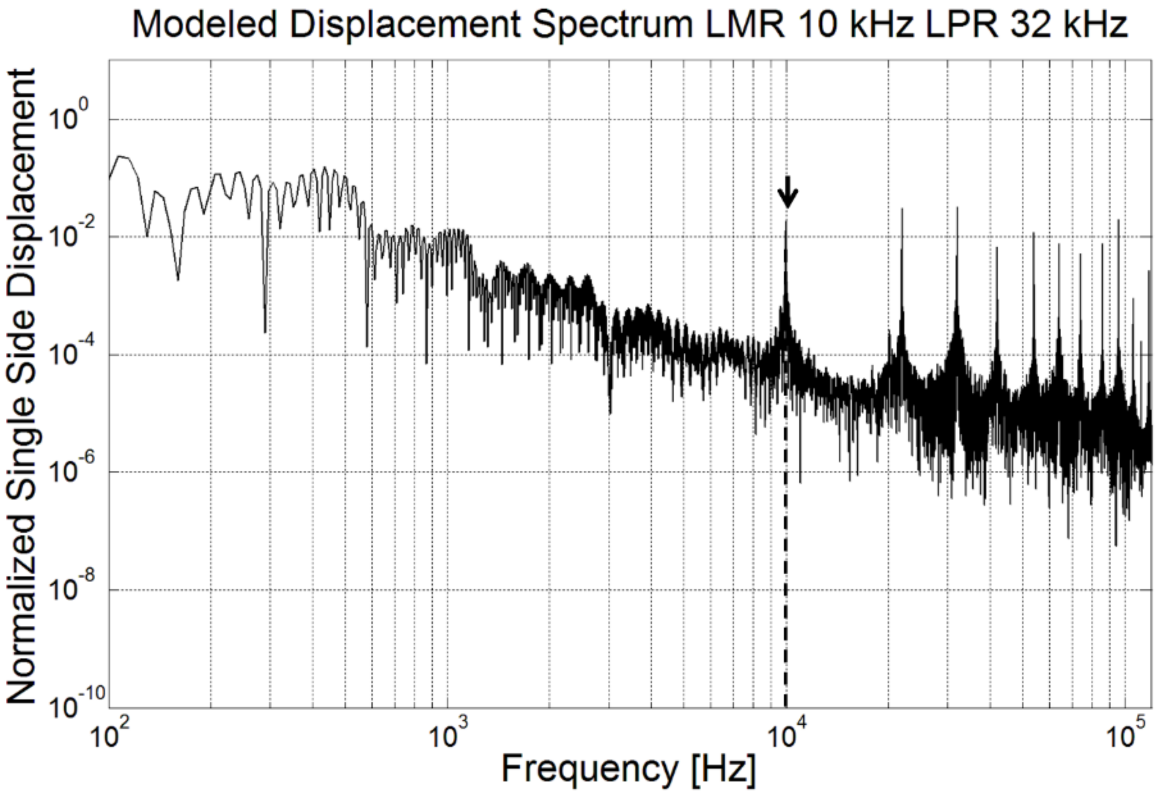

Modeled Displacement Spectrum LMR 10 kHz LPR 32 kHz
Normalized Single Side Displacement
10^0
10^-2
10^-4
10^-6
10^-8
10^-10
10^2
10^3
10^4
10^5
Frequency [Hz]

(k)

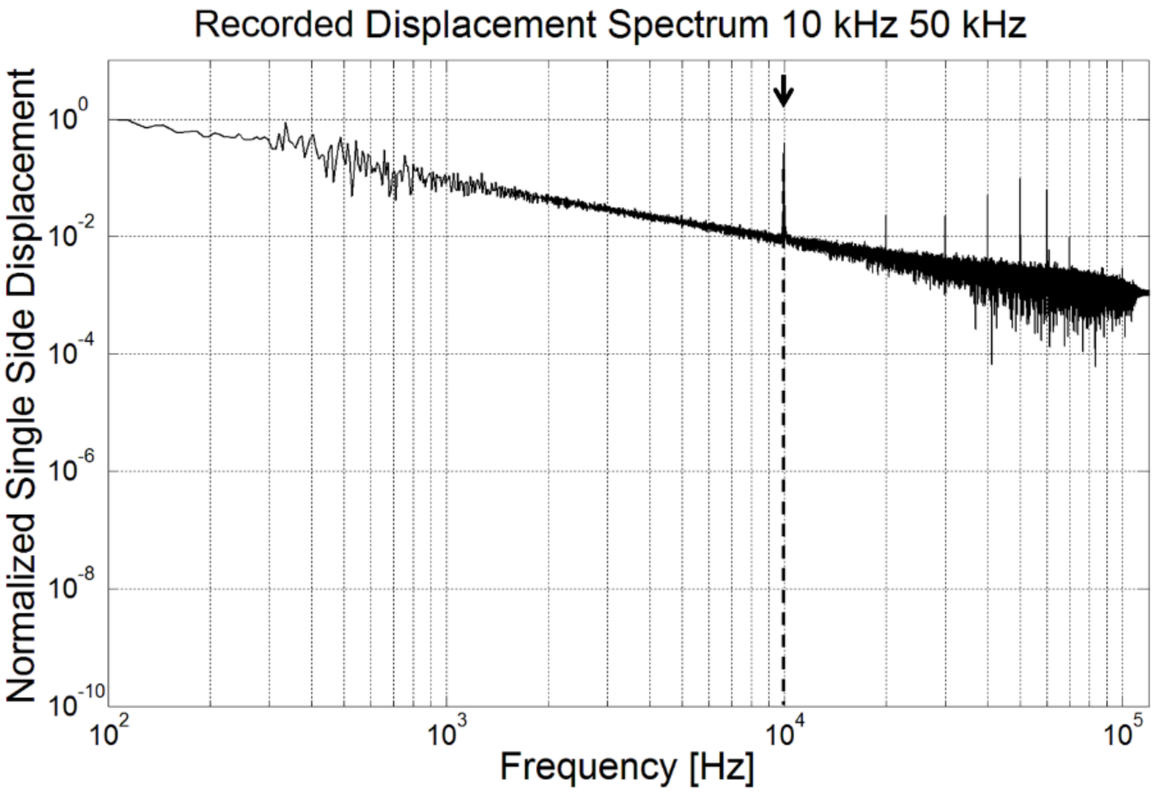

(l)

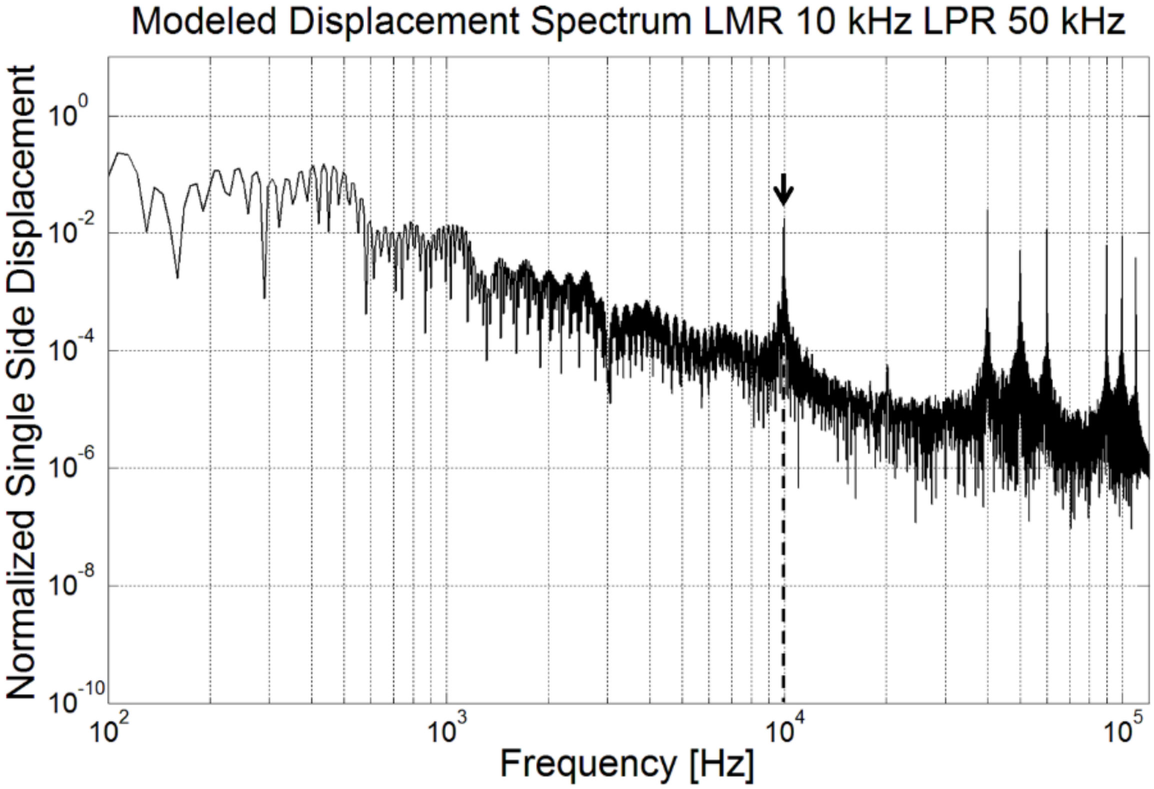

Supplementary Figure S2. In vitro normalized single sided displacement spectrum compared to the corresponding in silico (modeled) calculated spectrum for 1 kHz LMR and 32 kHz LPR (a-b), 1 kHz LMR/50 kHz LPR (c-d), 4 kHz LMR/32 kHz LPR(e-f), 4kHzLMR /50 kHz LPR (g-h), 10 kHz LMR/32 kHz LPR (i-j) and 10 kHz/50 kHz LPR (k-l). All spectra demonstrate a peak at the fundamental frequency f0 (black arrow) and an additional peak at the LPR. Modelled and recorded vibration displacements at the umbo for different LMR/LPR combinations demonstrate the ability to control frequency shifting through our optical monochrome

stimulation method. Additionally, the sidebands near the peak at the LPR demonstrate a characteristic pattern of pulse amplitude modulation.

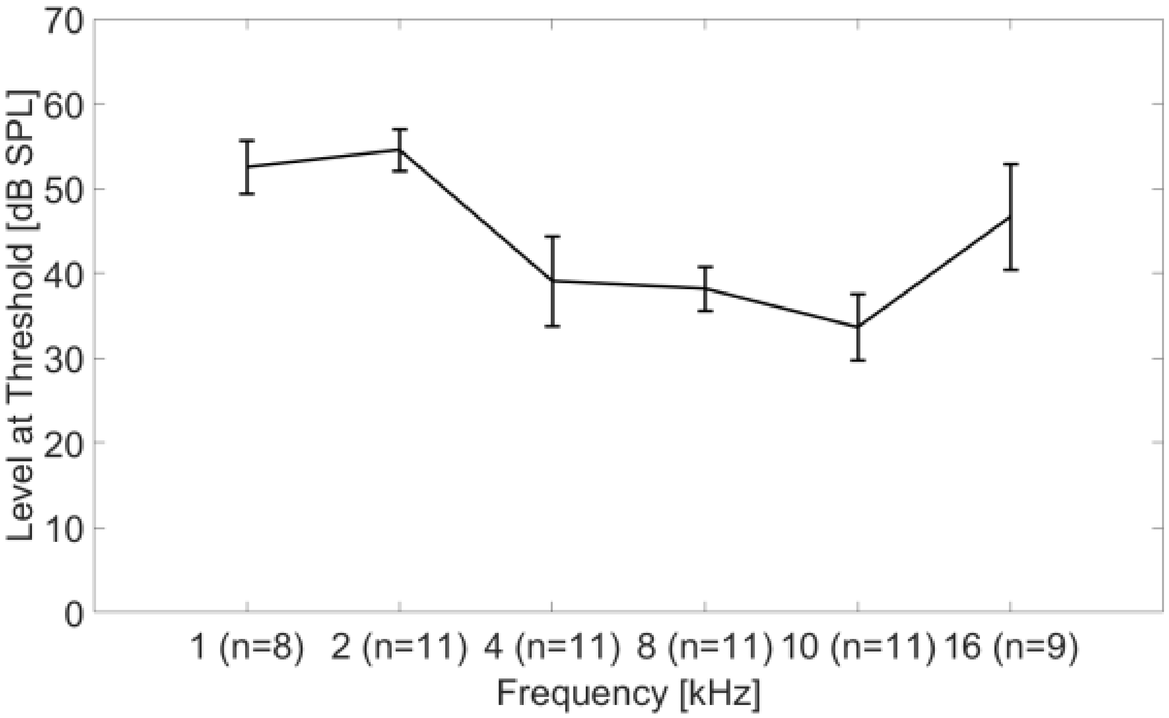

Supplementary Figure S3. Averaged acoustic thresholds determined from the aFRM at 1, 2, 4, 8, 10 and 16 kHz presented for the acoustic control measurements within the analyzed guinea pigs. Best acuity was around 10 kHz.

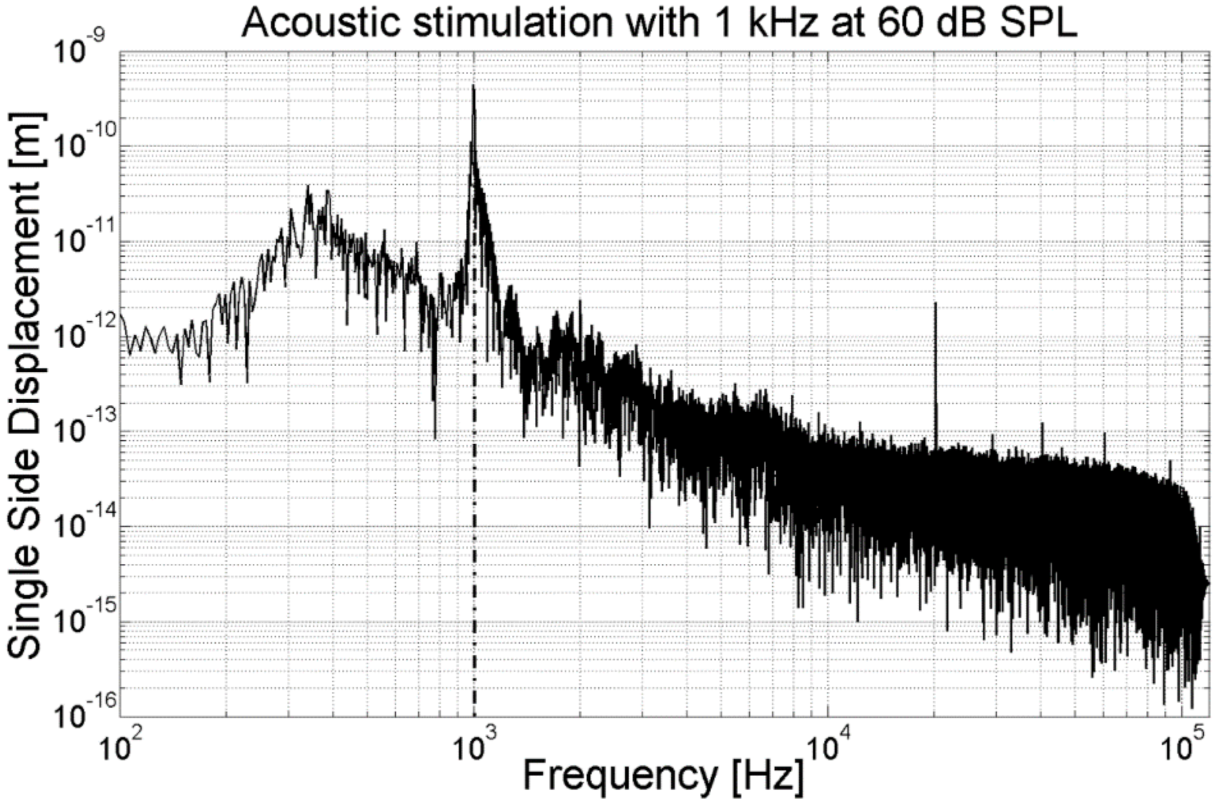
Acoustic stimulation with 1 kHz at 60 dB SPL
Single Side Displacement [m]
Frequency [Hz]
10^{-9}
10^{-10}
10^{-11}
10^{-12}
10^{-13}
10^{-14}
10^{-15}
10^{-16}
10^{2}
10^{3}
10^{4}
10^{5}

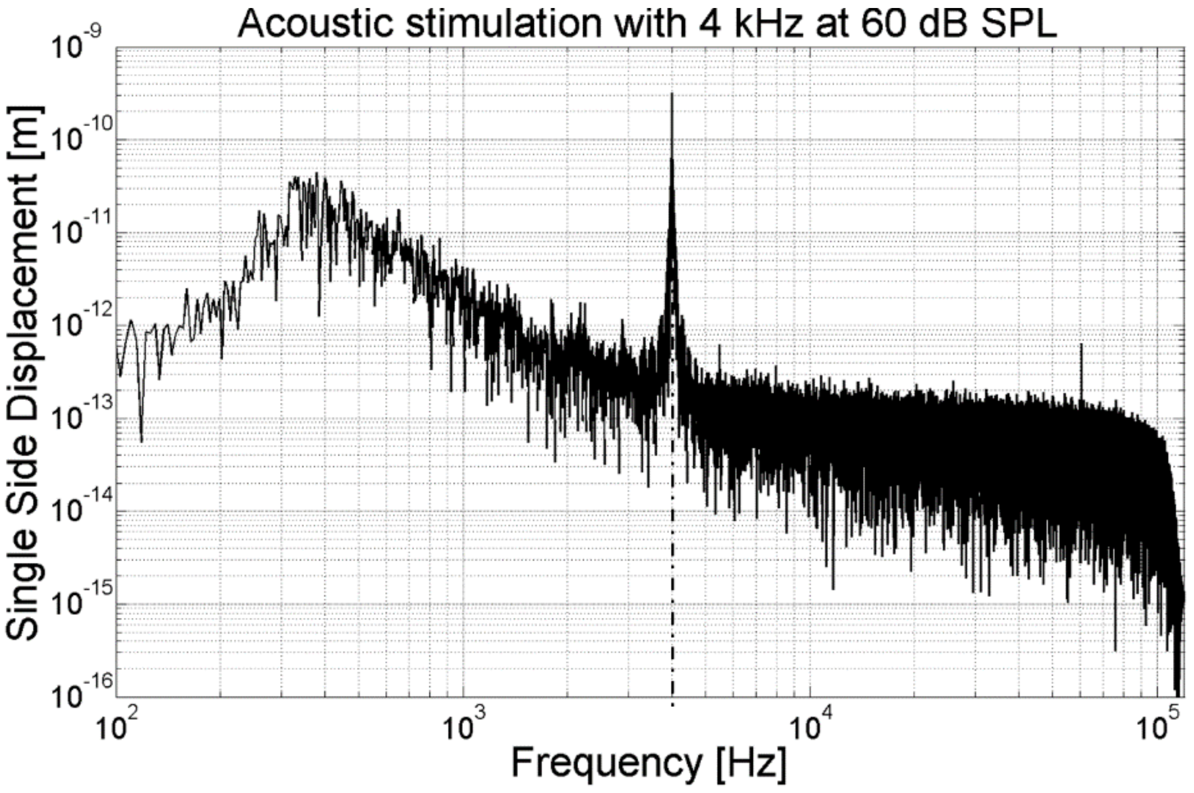
Acoustic stimulation with 4 kHz at 60 dB SPL
Single Side Displacement [m]
Frequency [Hz]
10^{-9}
10^{-10}
10^{-11}
10^{-12}
10^{-13}
10^{-14}
10^{-15}
10^{-16}
10^{2}
10^{3}
10^{4}
10^{5}

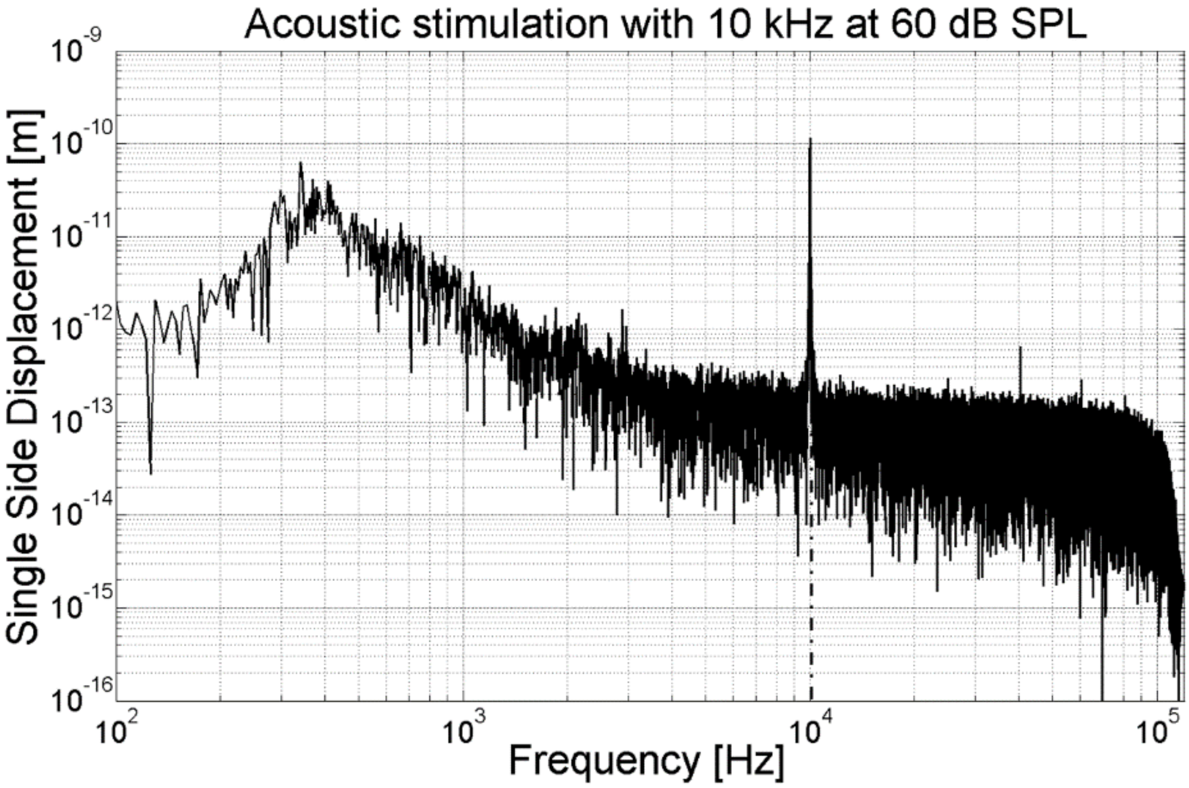

Supplementary Figure S4. Single sided displacement spectra after acoustic stimulation at the umbo with 1 kHz (a), 4 kHz (b) and 10 kHz (c). The peak at the fundamental frequency f0 (black dashed line) corresponds to the modulated stimulation frequency (LMR).

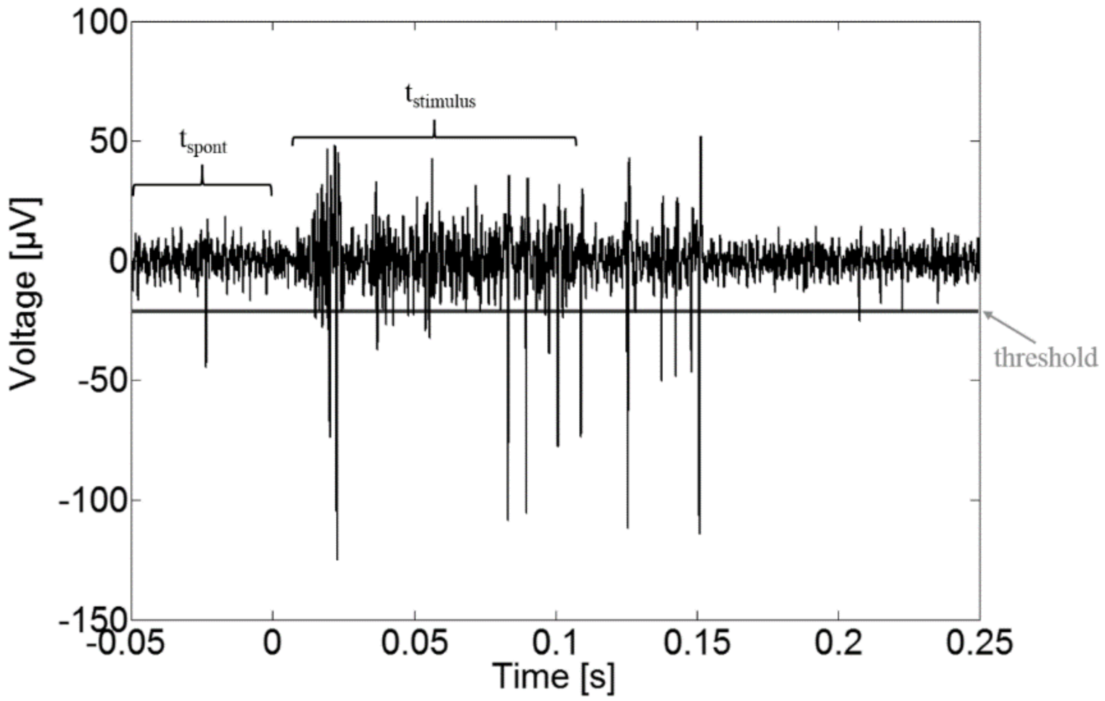

Supplementary Figure S5. Neural spike train recorded within the ICC in response to acoustic stimulation with spikes identified that crossed a specified threshold voltage level.

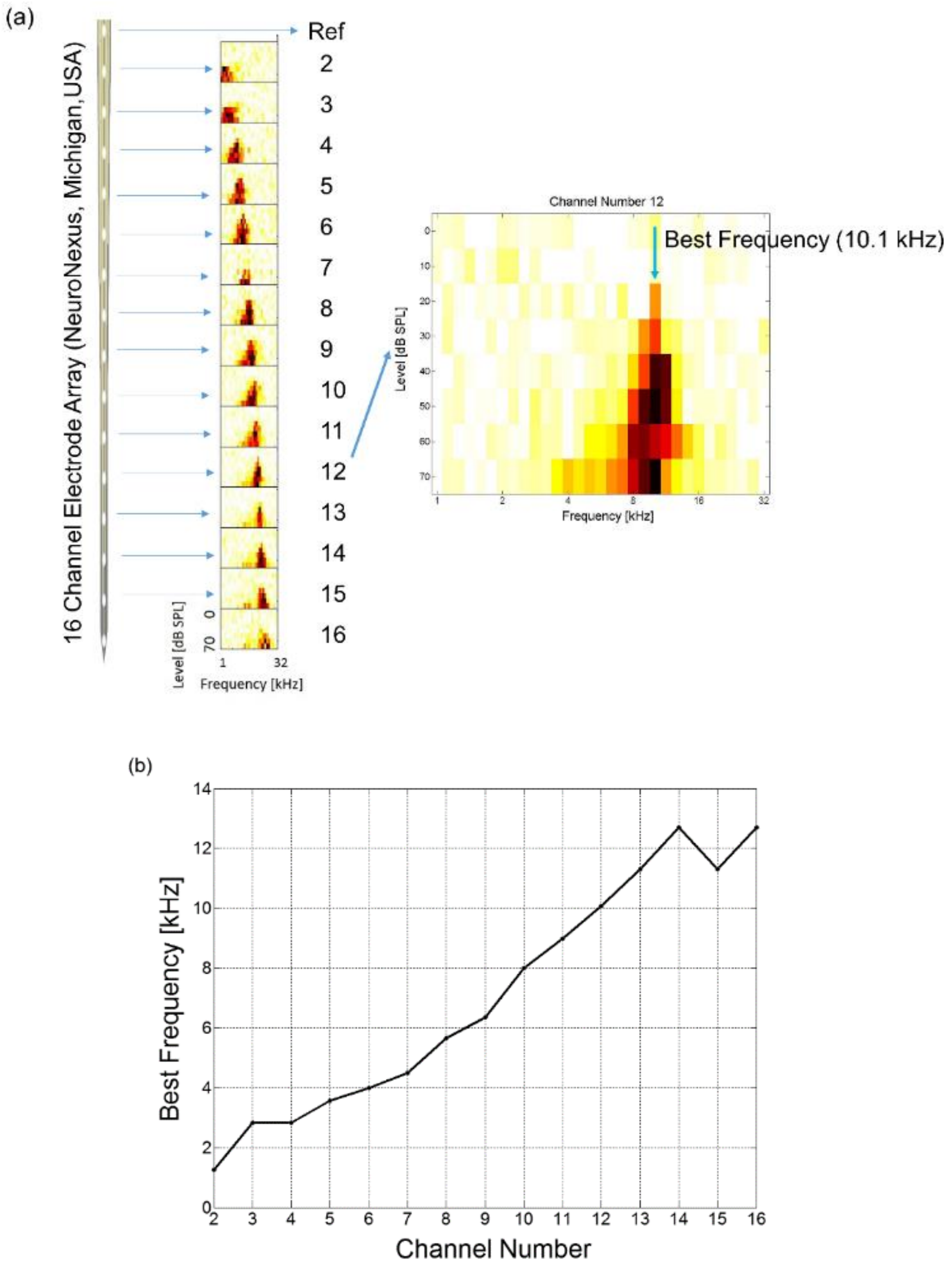

Supplementary Figure S6. (a) A1x16 electrode (NeuroNexus) with 16 channels, corresponding to the insertions depth of the ICC, with each box/pixel of an acoustic frequency response map (aFRM) consisting of the driven spike rate normalized by the maximum value for a given channel across all level and frequency combinations. The magnified example shows channel 12 corresponding to a BF of 10.1 kHz. (b) Example of a BF channel mapping with the channel number along the ordinate and the best frequency of each channel in kHz along the abscissa. The BF channel mapping demonstrates how the different electrode sites are positioned along the tonotopic gradient of the ICC.

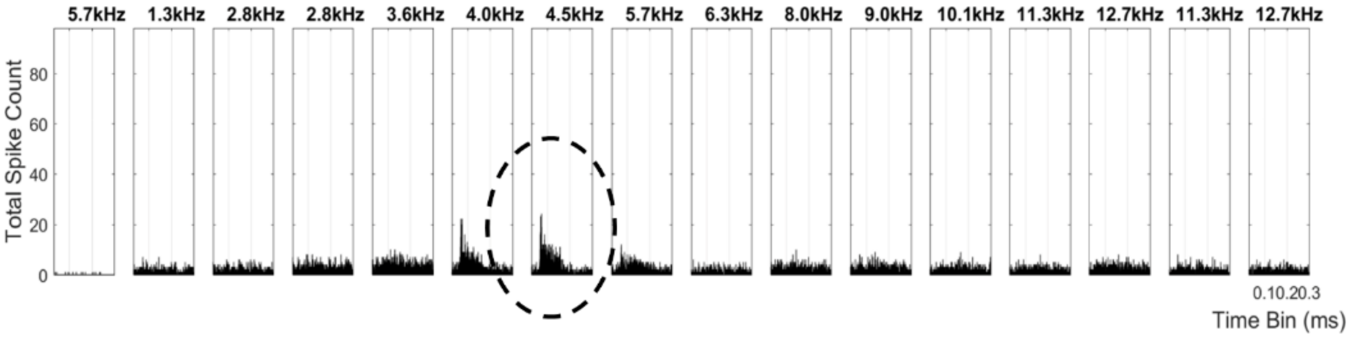

Supplementary Figure S7 Example of a PSTH in response to optical stimulation with 50 kHz LPR, 4 kHz LMR at an optical threshold of 0 dB LL (50 mW). Initial activity at threshold is observed on electrode sites near ICC neurons that have a BF near ~4 kHz, consistent with the stimulated LMR, demonstrating the frequency-specific stimulation capabilities of our laser modulation approach. PSTHs are based on at least 100 trials with 1-ms time bins.
